# Linen Most Useful: Perspectives on Structure, Chemistry, and Enzymes for Retting Flax

**DOI:** 10.5402/2013/186534

**Published:** 2012-12-30

**Authors:** Danny E. Akin

**Affiliations:** Russell Research Center, Agricultural Research Service, U.S. Department of Agriculture, Athens, GA 30606, USA

## Abstract

The components of flax (*Linum usitatissimum*) stems are described and illustrated, with reference to the anatomy and chemical makeup and to applications in processing and products. Bast fiber, which is a major economic product of flax along with linseed and linseed oil, is described with particular reference to its application in textiles, composites, and specialty papers. A short history of retting methods, which is the separation of bast fiber from nonfiber components, is presented with emphasis on water retting, field retting (dew retting), and experimental methods. Past research on enzyme retting, particularly by the use of pectinases as a potential replacement for the current commercial practice of field retting, is reviewed. The importance and mechanism of Ca^2+^ chelators with pectinases in retting are described. Protocols are provided for retting of both fiber-type and linseed-type flax stems with different types of pectinases. Current and future applications are listed for use of a wide array of enzymes to improve processed fibers and blended yarns. Finally, potential lipid and aromatic coproducts derived from the dust and shive waste streams of fiber processing are indicated.

## 1. Introduction of Flax and Linen Fiber

The history of flax (*Linum usitatissimum* L.) is long and important. The translation of its scientific name, “linen most useful” [1, 2], aptly describes its versatility and importance to world economy. Linen, the long, strong fibers from flax stems, is considered one of the earliest successes in textiles [3]. While evidence does not exist on how early people learned to separate fibers from the stems, flax as a major textile in ancient Egypt is well documented in depictions of its cultivation and processing [4]. Linen samples have been reported in the remains of Swiss lake dwellings dating back some 10,000 years [3]. Production and use expanded beyond the Mediterranean countries to central and northern Europe, making its way to Great Britain about 2,000 years ago from the Middle East by Phoenician traders [3]. Linen, as one of the primary fibers for Europe throughout the Middle Ages and the Renaissance period, was used extensively for clothing. Linen was important to Russia and its economy through various stages of its political history [5]. Flax became the greatest export item and the basis of economic life in Russia in the late 1800s and into the twentieth century. At one time, Russia produced about 80% of the world's fiber flax crop and before 1936 was the greatest exporter of flax. 

Fiber flax came to North America by European colonists. Production of flax in Connecticut was reported as early as 1640 [6]. While flax was grown in several regions of the United States, particular states developed well-organized commercial efforts, particularly Michigan and the Willamette Valley in Oregon [7]. The production of flax fiber in Oregon in the early 1900s, led by efforts of the US Department of Agriculture and the Oregon Agricultural Experiment Station, was remarkably well documented and included production yields, processing and mills, and other advancements [5, 8]. As in Europe, specially designed equipment to pull, turn, deseed, and scutch flax was developed to increase agricultural efficiency. Oregon's commercial enterprise for flax fiber, however, ended in the 1950s due to introduction of synthetic fibers and loss of government subsidies. 

The linen industry also declined in Europe due to the coming of synthetic fibers, such as nylon and polyester for apparel [3, 4]. Cotton before then, however, had overtaken the high position of linen and industrial flax fibers, which had existed for millennia, due to large amounts of inexpensive cotton and its improved mechanical processing. Generally, cotton has led as the natural fiber of choice since this time, with only short times of reversals such as blockades during the American Civil War (1861–1865) and disruptions caused by World War II (1939–1945). After the war, the lower production levels returned. 

In traditional linen-producing areas, promotional programs by linen industries in Northern Ireland and western European countries led to a strategic organization to promote linen in the 1960s [4]. In the 1980s, the FAO (Food and Agriculture Organization of the United Nations) sponsored workshops on flax, and in 1993, the “FAO Flax Group” became the “European Cooperative Research Network on Flax,” with coordination through the Institute of Natural Fibres, Poznan, Poland [9]. This program continues to compile data on crop production, facilitates interaction of several working groups, and sponsors numerous workshops thus promoting global interests in flax fiber [10]. Two publications per year still appear under its auspices. 

Recently, however, production of textile flax fiber has decreased in most of the reporting European countries and including the Baltic states and the Russian Federation [10]. In the European Union (EU) countries, production of flax declined from 122,379 ha in 2005/2006 to 73,029 ha in 2010/2011. Declining EU subsidies since the 1990s have reduced production levels and regions. Production in China, which has varied over the last two decades, has occupied a prominent position for the last several years [11]. However, the quality of Chinese-produced linen is not high enough for textiles, and China imports considerable amounts of long fiber from France and Belgium. The Belgium market for the prices of long fiber, which varies based on quality, recently was reported to range from 130 to 210€/100 kg, with prices for unscutched short fiber of 30 to 50€/100 kg [10].

After flax production ended in Oregon and effectively ended flax fiber production in the US, efforts to renew a flax fiber industry in the United States have occurred over the years. Most have not been successful. In 1998, Naturally Advanced Technologies (NAT) and the Alberta Research Council in Canada developed Crailar flax. NAT and the Hanes clothing company promoted the development of this product for complementary use with cotton in textiles. While the method is proprietary, popular news stories [12] and promotional websites [13] indicated that Crailar flax increased performance characteristics in textiles. 

Despite the reduction in production and usage from previous times, linen imparts characteristics of comfort, drape, and a distinctive appearance that have maintained a share of the market, particularly the luxury market for textiles [4, 10, 11]. Blending of cottonized flax, that is, short, refined flax fibers, with cotton and other fibers offers a potential for nontraditional, flax fibers to impart distinctive properties in textiles. For example, blending cotton with increasing levels of cottonized flax fiber for rotor spinning improved air permeability and wicking rates for moisture and modified the fabric structure [14]. The fine linen used for painting canvasses also requires strong and clean fibers from linen-type production systems, such as that from Belgium. Despite the reduced production of traditional linen and move to other sources, likely markets will exist for the long, strong, and clean linen fiber in specific, high-value applications along with cottonized flax fibers for blends [10].

The major source for flax fiber in North America, however, is straw from the linseed industry. Linseed provides an industrial oil widely used in paints, varnishes, cosmetics, and linoleum [15]. In the past and even more recently, flax seeds are being recognized as a health food, with the intact seeds providing a laxative effect and linseed products providing lignans and omega-3 fatty acids [16, 17]. It is likely, then, that production of linseed will continue and provide a consistent source of stems for flax fiber production. 

In contrast to agronomic systems to optimize fiber production in flax, linseed production seeks to optimize seed yield [18]. Short branching plants are grown until seeds are fully matured. Stems are thicker in these plants than in fiber-type flax plants. In North America, as well as Europe and Russia, linseed production is in colder climates, where fiber extraction from the stems is very difficult due to reduced microbial activities and poor retting (see later). Most of the North American flax fiber is extracted from linseed stalks by hammer-milling to obtain all the fiber, regardless of length, to be used in specialty papers. The quality of linseed fibers processed in this manner is considered too low for apparel or other high-value uses under the current commercial production and cleaning operations. Even with this hammer milled product for paper and pulp, however, there is currently interest in producing a cleaner fiber product, that is, less shive or nonfiber fractions, in order to reduce the amount of chemicals needed to remove lignin from the flax in pulping. 

Several research programs are in place to promote the use of flax and other natural fibers from various sources, including linseed stalks, in new products and in particular biocomposites. Efforts are currently under way to improve processing methods and find more uses for value-added fibers in industrial, for example, biocomposite, applications. In fact, industrial applications with composites and nonwoven materials may provide the greatest potential for expanded use of flax fibers in the future [19–21]. Canada, which is the world's leading producer of linseed [22], currently promotes expanding the flax industry, both for fiber and seed, through genetics research programs and organizations such as the Saskatchewan Flax Development Commission, the Composites Innovation Centre, and Biolin Research, Inc. in Saskatchewan, Canada [19]. To this end, Canadian research into flax has recently increased, particularly towards improving fiber quality of high-value applications such as biocomposites. North Dakota, the main producer of linseed in the US, follows the trend of the linseed industry in Canada.

About 20% of the straw from the linseed industry satisfies the specialty paper (mostly cigarette) industry in North America. The remainder of this straw by-product (greater than 1 million tons from western Canada) is now burned or chopped to spread on fields [22]. With the closing of the Ecusta operation, only one large processor for flax fiber from linseed straw, that is, Schweitzer Mouduit International, Winkler, Canada, still produces fiber and mostly for paper and pulp and lower-end uses. A very great opportunity exists with linseed straw to improve farm economy, replace synthetic fibers with natural fibers, and provide a value-added product for myriad applications [19]. Consistency in supply and in fiber characteristics must be addressed when flax fiber is sought for large-scale industrial usage. The degree of processing for fiber cleanliness will depend upon the end product desired, and for many products, the levels of cleanliness and processing costs are considerably less than for linen in textiles. With the North American textile industry based on short staple fibers (e.g., cotton), the possible use of new processing methods to provide textile-grade fibers from linseed straw should not be overlooked.

The goal will be to find improved ways of processing the fibers for consistent quality and with properties for applications in high-end uses, even for apparel [18]. Globally, an urgent and increasing need exists for new sources and products for agricultural, manufacturing, and health industries. While linen from fiber-type cultivars is still a valuable commodity for textiles, the desire for natural fibers in biocomposites points to linseed stalks as a largely untapped source of industrial fibers. Production of a higher quality fiber for value-added applications from the linseed stalks, however, requires some changes in fiber production and processing. Biotechnological approaches can supply innovative, efficient, and more directed methods for fiber production. 

## 2. Structure and Chemistry of Flax Stems and Relationship to Applications and Products

**Figure 1:**
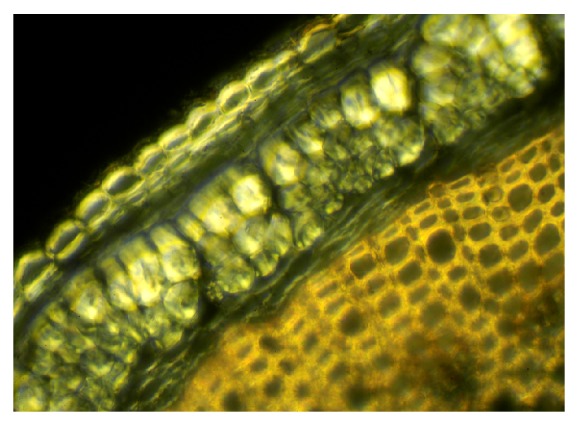
Free-hand cross-section of flax stem observed by polarized light microscopy showing the outermost cuticle/epidermal layer, birefringent fibers in bundles, and innermost core tissues.

The chemical composition and the anatomical location of constituents within the flax stem define processing, properties, and applications of flax. The anatomy of the flax stem and chemical composition in the cell types is well documented in many publications (e.g., [24, 37, 38]).  Figure 1 shows the arrangement from outermost to innermost layers as follows: cuticle connected with a single layer epidermis, bast fibers in cortical region, and woody core tissues. 

### 2.1. Cuticle and Epidermis

**Figure 2:**
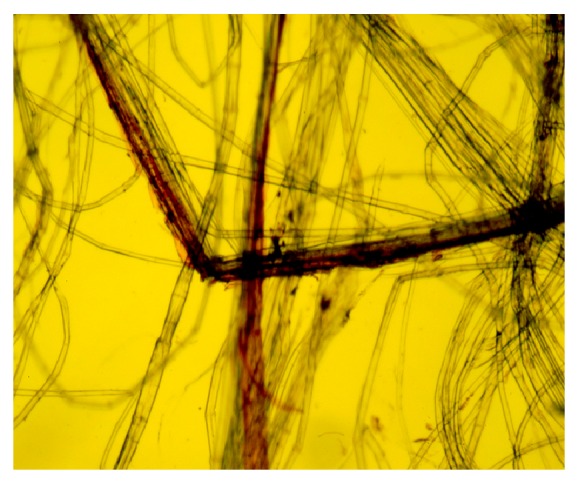
Light micrograph of processed fibers stained with oil red showing cuticle remnants still attached to the fibers.

The cuticle resides at the outermost part of the stem (Figure 1). Lipids, including waxes and cutin, and aromatics comprise this layer and provide a protective barrier to water loss and to invading microbial pathogens into the internal stem tissues [24, 25, 39]. The cuticle can be readily observed with the histochemical stain oil red [40, 41], which stains the wax in the cuticle a bright red color and thereby provides a distinctive, visible marker specific for the cuticle (Figure 2). Flax fibers do not stain with oil red, indicating no or a small amount of wax on fibers *per se*. By staining processed fibers with oil red, a quick, visible assessment of cuticle contamination is possible in “cleaned” flax fiber [25, 40]. 

The cuticle and the adjacent single layer of thin-walled, epidermal cells are closely connected, coming off as a unit (Figure 1). The “outer layer” of cuticle/epidermis comprises 13%–24% (by weight) of the bast fraction of selected fiber and linseed cutivars (Table 1) [23]. This layer, and mostly the cuticle, provides a rich assortment of lipids, including waxes, cutin, and sterols, while aromatics are present in small amounts (Table 1). Near-infrared Fourier transform Raman microscopy indicated, by absorption at specific wavelengths in stem thin sections, the presence of wax and soluble aromatic pigments in the cuticle [42]. Ultraviolet absorption microspectroscopy further indicated an absorption near 280 nm indicative of aromatics in the cuticle but not in the epidermis [24].

The parenchyma cells, including those below the cuticle/epidermis layer, lack aromatics and are a pectin-rich layer generally amenable to degradation by microbes and enzymes (discussed later), allowing separation of the cuticle/epidermis fragment during proper retting.

The cuticle is generally impervious to microbial attack as observed in field-retting of flax, although some disruption and penetration of the cuticle can occur by field-retting fungi [43, 44]. With pectinolytic enzymes, the cuticle can be sloughed off and freed from the fiber [45]. When this cuticle/epidermis fragment is intact, however, as when flax is underretted (Figure 2), it remains attached to several fiber bundles, resulting in large fragments and reducing the quality of fiber and yarn [25]. Total wax and cutin contents were significantly higher (*P* < 0.05) in commercial grades of low versus high quality fiber and yarn, likely because of the contamination by cuticle [25].

Cuticle, short fibers broken during processing, and woody core fragments comprise the dust particles that are emitted during fiber processing. The dust so generated is considerable, and dust bags or other suitable collection containers are used to maintain a clean environment. Potential lipid coproducts from the dust fraction, as well as from a purer cuticle fraction removed during experimental enzyme retting, have been evaluated [46, 47]. A hot alcoholic extraction was effective in removing the wax components, which could then be separated by controlled cooling [47]. These waxes and other lipids have potential as commercial products from this waste material of flax fiber processing. 

### 2.2. Inner Core Cells

The central woody core tissues are the primary xylem and other structural cells, which provide support and water conduction for the plant (Figures 1 and 4). The core cells are about 65%–75% of stem material. The main sugars are glucose, representative of cellulose, and xylose, representative of hemicelluloses (Table 2) [24]. Other carbohydrate components are lower in amounts and represent hemicelluloses and pectins. 

Almost all of the lignin in flax stems is present in the core cells. The histochemical stain for lignin, acid phloroglucinol [48], stains the entire core cell walls a bright red color, indicating coniferyl-rich constituents throughout this tissue (Figures 4 and 5). Positive reactions with acid phloroglucinol for coniferyl lignin (monomethoxylated aromatic rings) and chlorine-sulfite for syringyl lignin (dimethoxylated aromatic rings) [48] indicated that both lignin types are present in core cells. Lignin values for core cells have been reported at 25%–30% [49]. Results, however, vary with the analytical method used, such as Klason lignin with 72% sulfuric acid or that derived from permanganate oxidation. Often, Klason lignin gives higher values because components other than aromatics are in the residue. By alkaline extraction and subsequent summation of similar structures, specific lignin types were determined. Using this method, aromatic levels totaled about 1.5%, with a higher level of guaiacyl to syringyl units (Table 3). The low S/G ratio of lignin components was also shown by others [49]. Of the low molecular weight aromatics, only small amounts of ferulic acid were found (Table 3) [24, 49]. Nuclear magnetic resonance (NMR) spectrometry confirmed that the aromatics were present as lignin [24]. Further, ultraviolet (uv) absorption microspectrophotometry of thin sections also showed a strong absorbance near 280 nm in the core cell walls and no absorbance beyond, but absorbance maxima shifted slightly in cells more towards the stem center [24]. The presence of a single strong absorption near 280 nm, as well as lack of absorbance near 320 nm, suggests a complex structure of the lignin similar to that in woody plants [50]. 

During field retting by indigenous fungi, the core cell walls remain intact and are virtually impervious to fungal attack, showing a typically high resistance to degradation for this type of lignocellulose [24]. These lignin-rich core cells make up a large portion of the “shive” waste fraction generated during fiber processing. With such a high proportion of stem material, shive waste is a huge by-product of commercial fiber production. Unless there are uses for it, shive becomes an economic deficit in processing, and its disposal is necessary. Shive, however, has economic value in the overall flax processing system, generally now finding application in low-value uses typical of lignocelluloses, such as animal bedding, mulch, particle boards, and thermal energy from burning. These additional sources of revenue provided by selling the shive, 9.00€/100 kg [10], are essential to guarantee a positive economic position of the flax processing facility. More recently, the lignocellulosic nature of shive has shown to be exceptional as activated carbon for heavy metal absorption [51] and for removal of chlorinated hydrocarbon [52], outperforming selected commercial products. Extraction and separation of cellulose, hemicellulose, and lignin from flax shives by various methods, notably pressurized low polarity water extraction and under different conditions of pH and temperature, are reported as effective means of obtaining aromatic feedstocks from this by-product of fiber processing [49, 53]. 

The fact that the core cells, that is, shive, contain by far the most aromatics in the plant stem while fiber contains very little gave rise to a rapid method to assess fiber cleanliness. Near infrared reflectance (NIR) spectroscopy coupled with chemometric models for ratios of shive : fiber from 0% to 100% was used to develop a method to predict shive content in cleaned fiber [24, 54–57]. This method has been further developed and employed for at-line assessment of quality in which commercial bales were assessed over several weeks [58]. In converse, the cleanliness of shive from residual fiber left from processing can be similarly predicted in materials sought for clean woody lignocellulosic materials [59]. 

### 2.3. Bast Fibers

The industrially important bast fibers are long, slender, and strong specialized cells that develop in bundles in the cortex region, located between the cuticle/epidermis layer and the innermost woody cells (Figure 1). These cellulose-rich cells are the source of linen and other commercial fibers. Based on quality properties such as length, strength, and cleanliness, the bast fibers are sought for high-value apparel and other textiles, natural fiber composite reinforcement, and specialty papers such as cigarette, currency, and Bible sheets [15, 18, 19]. 

In nature, these fibers exist in bundles of individual (or ultimate) fibers encircling the lignified core tissues (Figure 1). Ten to 40 spindle-shaped ultimate fibers, each 2 to 3 cm long and 15 to 20 *μ*m in diameter, form in 20 to 50 discrete bundles [3, 37]. Nodes or “fibernodes” [60], which are dislocations perpendicular to the axis, occur in fibers and entire bundles, as clearly shown by microscopy (Figure 6). What creates fibernodes is not clear, and kink bands are similar in structure and apparently give similar responses. Possibly, pressure exerted during mechanical handling or even through growth and expansion of tissues may create their presence. These fibernodes are important, because dyes, enzymes, and other liquids preferentially react there first [61–63]. Cellulases also attack preferentially at the fibernodes, even across bundles (Figure 7). Breaks during stress and compression tests occur preferentially at fibernodes and kink bands [64, 65]. 

Cellulose is the main component in bast fibers, with values of 65%–80% of the dry weight reported [63]. In addition to cellulose, bast fibers contain pectins, hemicellulose, and aromatic compounds in small amounts (Tables 4 and 5). Field-retted fiber showed an expected increase in glucose (by weight) indicative of cellulose, while increases also occurred in mannose and galactose. These noncellulosic sugars appear to be inherently part of the fiber [66]. The fact that hemicelluloses, such as galactoglucomannans and xylans are substantial components in flax fibers has been shown by considerable research [63, 66–68]. Distinguishing characteristics of linen, such as high moisture regain, may be influenced by the presence of these noncellulosic carbohydrates within the cellulosic structure. Proteins and proteoglycans are also associated with secondary walls of flax fiber and possibly provide structure [69].

Data on lipids associated with bast fibers were collected from several studies and presented in Table 5. Fibers that had been manually separated and cleaned of all other visible materials showed the presence of low levels of waxes, cutins, and sterols, with amounts of about 0.2% of fiber dry weight and 1/20th or less of levels in the cuticularized epidermis. Other work, however, on a series of dew-retted and water-retted flax fibers from Europe [27] showed that lipids were present on fibers, with higher levels on the better quality (i.e., stronger, and finer) water-retted ones (Table 5). With these latter samples, it was not clear if the lipids represented residual cuticle or if these fibers had more inherent lipids on the surfaces. Chemical analyses of highly cleaned fibers from linseed flax and mature plants (i.e., grown for seed) had higher lipid levels than did bast fibers of the fiber-type plants (Table 5). These compounds in linseed may reflect a higher level of waxes on the fibers or may reflect more difficulty in removing all the cuticle from fibers during processing. The use of oil red as a histochemical stain, however, did not indicate wax on the clean fiber surfaces, and so likely it is residual cuticle (Figure 2). 

**Figure 3:**
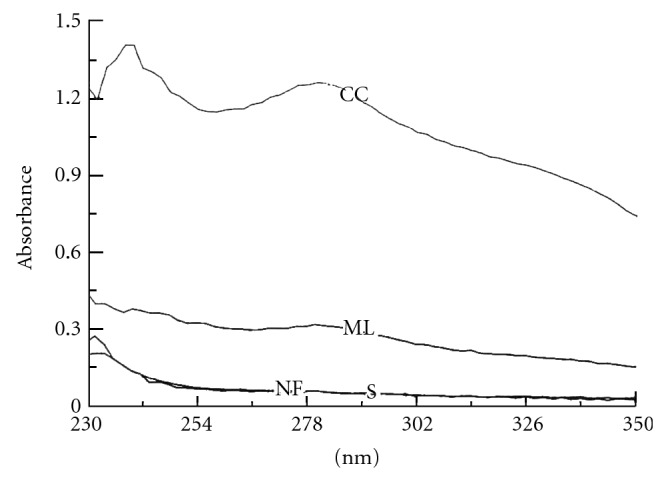
Ultraviolet microspectrophotometry of thin section of unretted flax stem showing the absorption over a range of 230–350 nm. Spectra CC is of selected cell corners of fiber bundles, ML is middle lamellae, NF is nonfiber region, and S is secondary wall of bast fiber. From [24].

**Figure 4:**
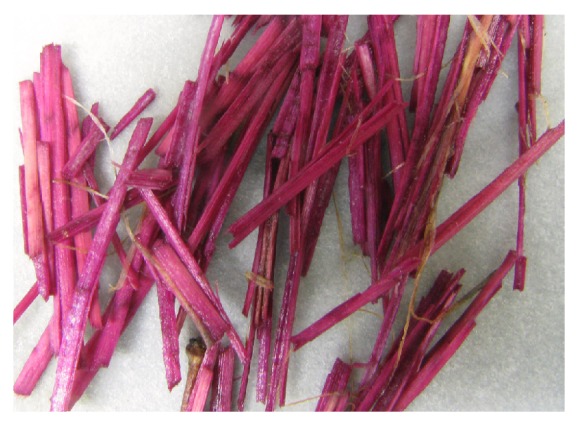
Digital photograph of the stem fraction separated from fibers during processing and stained with acid phloroglucinol. The stems are bright red and well differentiated from fibers.

**Figure 5:**
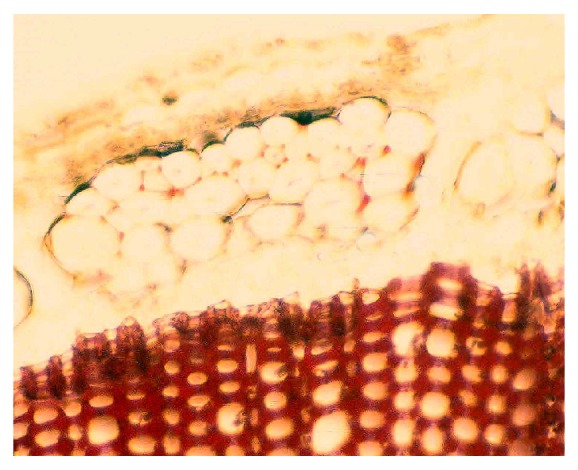
Light microscopy of thin section of flax stem stained with acid phloroglucinol indicating lignin throughout all the core cell walls. Fibers do not stain with acid phloroglucinol, except for a few cell corners.

**Figure 6:**
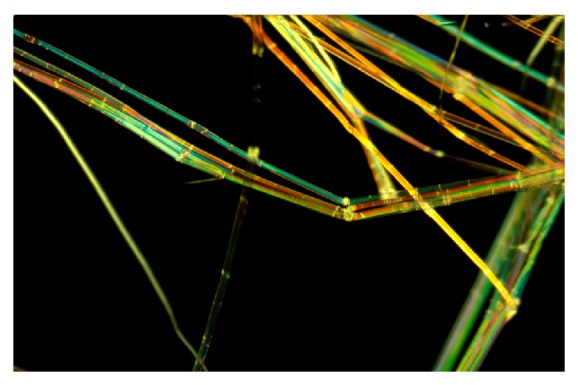
Polarized light microscopy of flax fibers showing bending at fibernodes, with nodes and kink bands across several fibers.

While the question of lignin in bast fibers frequently arises, most data indicate that the amount of aromatics present is small [23, 24, 28]. Localization of aromatics, using histochemical stains [24, 70] and ultraviolet (uv) absorption microspectrophotometry [24] showed that aromatic compounds were limited to middle lamellae and cell corners in bundles, and by far, the greatest levels were in cell corners (Figures 3 and 5). The deposition of aromatics, however, as shown by both methods, was sporadic in the bundles. Furthermore, staining with acid phloroglucinol [48] suggested a coniferyl-type lignin. Other work, however, using solid phase ^13^C NMR (nuclear magnetic resonance) spectrometry indicated that aromatic material in flax fibers was predominately an anthocyanin, rather than lignin [71]. Spectroscopic analysis of water-soaked, manually-separated, and then enzyme-retted fibers, which were free of all nonfiber materials, indicated only trace aromatics in fibers from fiber- and seed-type flax stems [72]. So, while trace amounts of aromatics are found in fiber bundles, the amount is small and does not appear to impede fiber/core separation in either fiber-type or linseed straw [28]. Often pockets of acid-phloroglucinol staining are observed on fibers, without the presence of obvious shive material, and likely represent a residual from either core or cuticle or the aromatic material from cell corners. Possibly, heavily localized areas of aromatics that remain on retted fiber could influence fiber quality properties [73] or reduce processing efficiency. 

Extraction of flax bast tissue, which included fibers and cuticle/epidermis, with a series of organic solvents (i.e., hexane, propanol, methanol, and water) and analysis by reverse phase high pressure liquid chromatography (HPLC) and ^13^C NMR indicated a variety of aromatic constituents including flavonoids and hydroxy-methoxy cinnamic acids [74]. The water extract from these flax samples contained a complex mixture of compounds, including sugars and aromatics. The phenolic-containing extracts inhibited cellulase and pectinase activities, suggesting a possible influence on retting enzymes if such compounds were released. Based on the previous discussion, the most likely source of these aromatic compounds in this study was the cuticle of the bast layer, rather than the fiber.

In the cellulose of plant cells, generally, a structure of unbranched linear glucose units allows zones of higher order, that is, crystalline regions, as well as areas of lower order, that is, noncrystalline regions [75]. In flax fibers, X-ray diffractometry shows region of crystalline and less-ordered structure, with the linear orientation of both regions higher in flax than the other cellulosic fibers cotton and ramie [63]. The secondary cell walls of flax fibers at maturity are reportedly locked in an almost axial direction [66], giving lower elongation and a more brittle nature for flax fibers compared to cotton fibers. While flax fiber is primarily a cellulosic fiber, its chemistry and characteristics provide specific properties that differ from cotton and many other natural fibers. 

### 2.4. Other Cell Types and Structures

Parenchyma, cambium, and the middle lamellae, which bind ultimate fibers in the bundles, are particularly rich in pectins, hemicelluloses, and other matrix polysaccharides as shown by response of these tissues to pectinolytic enzymes [26]. Lignin is lacking in these structures for the most part. The separation of fibers from the woody core occurs at the outer surface of the cambium and is facilitated when stems have been stored in dry climates for an extended time. Proper retting also releases the cuticle/epidermis layer from the fiber bundles. It is the pectin-rich regions that are of prime importance in retting, and considerable work has been done on the pectinases and ways to degrade the pectin in flax. 

### 2.5. Pectin

Pectin is a complex polysaccharide of many plant cell walls and plant tissues [76]. Pectin, while often low in amounts, is strategically located and binds cell walls within plants [66]. While pectin is, therefore, particularly important in maintaining the structure of flax stems, its degradation is of fundamental importance for retting and the resulting quality of flax fibers [37, 73]. 

Chemically, pectin is a heteropolysaccharide consisting mainly of 1,4-linked *α*-D-galacturonic acid, with various degrees of methylesterification at the carboxyl position and with various attached side chains [77]. In some cases, pectin in primary plant cell walls may have a high proportion of oligosaccharide chains on the backbone and longer chains than the pectin in the middle lamellae [77]. NMR spectrometry indicated that a rhamnogalacturonan structure of type I pectin, which is a prominent form in plants, likely forms the backbone of the high molecular weight polysaccharides in flax fiber [78]. In retting of flax, pectin degradation was reported to be faster in flax harvested during flowering than in mature flax stems, and a residual pectin level of 7 to 10 g/kg remained after retting [79]. 

Chelators to remove Ca^2+^ or other divalent cations are known to improve retting. Sharma patented a chemical retting process using ethylenediaminetetraacetic acid (EDTA) [80]. Nonmethoxylated carboxyl groups on galacturonic acids are often cross-linked by Ca^2+^  to form stable bridges across pectin molecules [77]. Mid infrared microspectroscopy mapping of mature flax fiber indicated that pectin types varied among plant types and regions [81], with the potential to influence retting efficiency. Immunocytochemical staining methods, using gold-labeled antibodies against specific pectin structures, provide further indications that the sites of specific pectin types vary within areas and even layers of flax fibers [82, 83]. Both nonmethoxylated pectin and calcium levels are higher in the epidermal regions of the flax stem and lower in the fibers [84]. Inductive coupling plasma (ICP) emission spectrometry showed that calcium levels in the cuticle/epidermis tissue was 5.5-fold greater than in the bast fibers of “Ariane” flax [65]. High amounts of calcium in the rigid cuticle/epidermis fragment further stabilize an already formidable barrier to retting in flax stalks. Endopolygalacturonase, a pectinolytic enzyme that is present in many enzyme mixtures, was reportedly inhibited by steric hindrance through calcium linkages in pectin [85]. 

The reported levels of pectin in flax vary considerably and are influenced by various factors [86–88]. For decorticated, that is, processed, flax fiber and cell walls of various cultivars, the pectin content ranged from 20.5% to 34%. Chemical treatment with dilute hydrochloric acid followed by ammonium citrate resulted in a pectin content for flax fibers of 1.6% [88]. While only an approximation, the sum of uronic acids, rhamnose, and arabinose was 1.7% of the total carbohydrates for field-retted Ariane flax fiber [24].

## 3. Retting of Stems for Fiber Extraction

To obtain bast fibers for commercial use, flax stems undergo a process called retting to separate fibers from nonfiber materials, namely, cuticle/epidermis and the woody core. The method first used for separating and cleaning linen fibers is not known and was “possibly an accidental observation of the fact that flax stems…turned to fibers under certain conditions of exposure to weather or immersion in water…” [4]. The historical value for this information has importance in some contexts, and searches are underway to discover the method early linen producers used to produce their fine strong fibers for textiles. 

**Figure 7:**
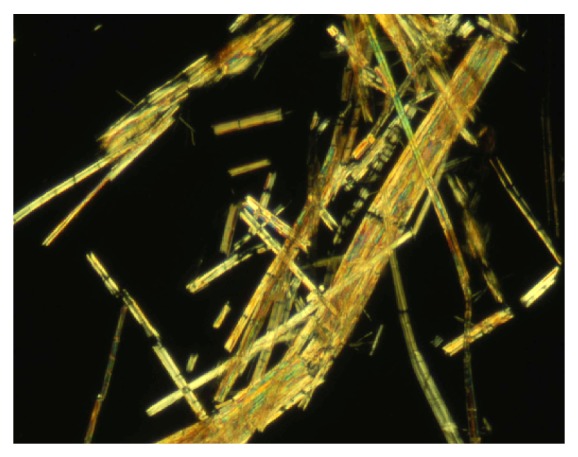
Polarized light microscopy of commercial flax fibers incubated with cellulase showing preferential attack at the fibernodes and kink bands.

Retting is primarily a microbial process. The main idea is to degrade the pectins and other cementing compounds that bind the bast fibers and fiber bundles to other tissues and thereby separate fibers from nonfiber materials [3, 18, 37]. The separated fibers are then cleaned of nonfiber materials by mechanical processing [18]. For long fibers used for linen, specialized equipment is employed; the first stage is called scutching [89], which uses a specialized system to beat and stroke long fibers to remove shive, and the second is hackling [90], relying on a specialized instrument to comb, straighten, and align fibers. Insufficient retting, or under-retting, results in poor separation of the cuticle/epidermis layer and the woody inner tissues (i.e., shive) from the fibers [25, 31]. Subsequent cleaning is then problematic, because the nonfiber materials become entangled in the fibers and reduce fiber yield, processing efficiency, and ultimate fiber quality. Conversely, overretting can occur. In this situation, the cellulosic fibers are weakened by overly active cellulases (Figure 7), resulting in poor fiber quality. Retting is of ultimate importance in fiber yield and quality. Even though currently most of the linseed straw fiber is of fairly low quality and used for paper and pulp, a desire exists to have less shive in the hammer milled fiber material in order to reduce chemicals used for delignification in making pulp. The fact that linseed straw is produced in regions unsuitable for dew retting is problematic in having high fiber yields and clean fiber. 

Two main methods have been historically employed commercially to ret flax for textile-grade fibers, namely, water retting and dew (or field) retting [3, 37]. In traditional water retting, as practiced in western Europe when the linen industry was flourishing, flax stems were pulled and submerged in bodies of water, for example, lakes, rivers, and ponds, for five to seven days. Afterwards, retted stems were dried and sun bleached in the field. Anaerobic bacteria, primarily pectin degraders, were the main organisms in water retting. Because the process was understood to some extent, technological methods were adapted to improve the process. Retting pits or tanks were constructed where temperature could be controlled. Selected microorganisms were chosen to improve water retting. At times, tanks were aerated to modify the bacterial consortium and thereby the bacterial metabolism, reducing the problems of pollution and stench from anaerobic metabolism that caused widespread concern in areas of western Europe [37].

 Water retting resulted in long, strong, and fine fibers of excellent quality for apparel and other textiles. The high cost of this method and resulting pollution, with the stench residing in water-retted fibers, however, resulted in water retting being abandoned for the most part in the mid 1950s in western Europe [37]. Water retting has been mostly replaced by dew retting, although some water retted flax was commercially available in the early 2000s [91]. Reports indicate that China, the largest producer of flax, may still produce water retted fiber in aerated tanks, but the fiber quality is not reported to be of good quality for textiles [11].

Dew (or field) retting is the method used in western Europe for obtaining high quality fibers for textiles. Field retting, however, is reported to be the oldest method of retting flax, practiced thousands of years ago by the Egyptians [37]. Field retting is carried out by pulling flax stems and laying them in even layers of rows for the moisture to encourage indigenous fungi to colonize and grow on the stem. The farmers of western Europe reportedly produced the best dew-retted fiber because of the climate and their knowledge of when to turn and harvest the flax for uniform retting. In areas of proper climate and expertise, commercially dew retting works and has been the method of choice for linen and other flax fiber production. Most of the world's textile flax fiber is produced by field retting [10].

The quality of flax fiber has declined over the years since dew retting replaced water retting as the main method for getting textile grade fibers in western Europe [92]. In addition to lower quality, field retting results in an inconsistent quality fiber. Dew retting continues to be the main retting method over water retting because production costs are lower, however, and fiber yields are higher and there is no stench. Dew retting, though, has a number of disadvantages other than poor and inconsistent quality compared to water-retted fiber. Certain areas formerly known for their linen production are unable to ret because of the noncompliant climates, such as England, Scandinavia, and Ireland. Field retting only works with appropriate moisture and temperature for fungal activity. Another disadvantage of field retting is that large tracts of land are tied up for weeks until flax is suitably retted. In intensive agricultural areas (e.g., for multiple cropping), farmers are disadvantaged by having land occupied by retting flax. Dew-retted fiber is dirty due to the fungi and soil. The vagaries of weather constantly threaten the harvest. Too dry weather results in poor fungal growth and lack of proper retting; wet weather delays field harvest and also interferes with fungal growth, resulting in pockets of anaerobic degradation. Over-retting occurs with excessive growth of cellulolytic fungi in the retting consortium and results in weakened fiber. So, even in the best regions for field retting, crop losses of about one-third are expectedly for one reason or another. 

From the early 1900s, technological efforts have been attempted to improve retting [37]. This subject has been briefly and recently reviewed by this author [18, 38]. Methods for improved retting included modifying water retting or dew retting to remove the inherent problems and to select specific retting microorganisms. Stand retting, where standing plants are dried with a herbicide, notably glyphosate (N-phosphonomethyl glycine), and allowed to ret by indigenous microorganisms in a modified form of field retting, has been tried [93, 94] and is still being developed [95]. Often the fiber properties were shown to be improved over dew retting, although dry weather interfered with retting and was problematic with this herbicide [96]. This method is still promising, particularly with new forms of the herbicide, and has been used to produce test plots of flax for cottonized fiber in England [97].

In addition to modification of the traditional water and field retting methods, much research has focused on chemical retting approaches, sometimes with and sometimes without microorganisms or enzymes [80, 98–106]. As mentioned previously [80], the use of chelators, notably EDTA, has been pursued, and its value is shown by strong sequestration of Ca^2+^ at various pHs [98] to disrupt the pectin linkages. Autoclaving flax straw with the chelators EDTA and oxalate has been used with breeding programs to effectively extract unretted flax fibers [101]. A patent exists for a mechanical process to produce fiber strips followed by a chemical/cooking process under pressure [102]. Flash hydrolysis or steam explosion treatment, with or without impregnation before steam treatment, has been used to remove pectins and hemicelluloses from decorticated flax to produce small bundles and ultimate fibers [104–106]. Ultrasonic treatment, following decortication and opening of green flax or hemp stalks, has been used to obtain fibers from diverse sources without the use of chemicals [103]. The use of low energy, uniform ultrasonic treatment, combined with enzymes, has shown increased activity of various enzymes for cotton fabrics [107] and could be a useful method to improve the efficiency in retting flax. Chemical separation has resulted in successful laboratory results, but at times, fiber properties are less satisfactory than those from other methods. Efforts are reported to be still underway to assess physical and chemical methods to separate fiber. The use of enzymes, focusing on pectinases, also has been researched for some time [37, 108–110]. None of these methods has replaced field retting as a commercial practice. Enzyme retting, however, has proven to offer promise as a biotechnological improvement and is still undergoing research and development for improving fiber quality.

### 3.1. Enzyme Retting

Sharma and his colleagues in Europe and the United Kingdom in the 1980s carried out a major effort on enzyme retting, primarily attempting to mimic water retting with a consortium of plant cell wall-degrading enzymes. These efforts, as well as other topics related to flax production for textiles, have been documented in numerous papers and in the book *The Biology and Processing of Flax* [111]. Since the plant cell wall is a complex lignocellulosic material, it was believed that a mixture of cellulases, hemicellulases, and most notably pectinases was required to ret flax as occurred with the plant-associated natural microbial consortia in water or dew retting [110]. In hindsight, part of this approach could have been due to the lack or cost of specific enzymes available at the time, as shown by attempts to use highly pectinolytic microorganisms [110]. Fungal cultures contained this mixture of cell wall-degrading enzymes but with some different profiles and activities. Microorganisms with high levels of pectinases were chosen, but most culture filtrates also contained some cellulases.

 Sharma and colleagues enjoyed success in their work. Several commercial enzyme mixtures containing plant cell wall-degrading enzymes were tested. A product from Novo Nordisk (Copenhagen, Denmark) called SP 249 was used in a side-by-side test of enzyme versus water retting [108]. SP 249 was used at 3 g/L in an 11 : 1 liquid-to-solid ratio at 45°C for 24 hours. Eighty kg of flax stalks were submerged for each of the two test methods. After retting, fiber yield and quality were equal for the two retting procedures. Oxidizing agents, however, were required to denature the enzymes and stop the continuing action of cellulases in the enzyme mixture. The study showed the successful application of enzyme retting in pilot plant scale, promising that retting of flax could occur with enzymes and with a reduced time of retting [108]. The liquid method also ensured a more consistent product and could be carried out in any location with proper equipment. From the work of Sharma and colleagues, Flaxzyme, which was a patented liquid preparation of balanced cellulases, pectinases, and hemicellulases from *Aspergillus *species, was developed by Novo Nordisk (Copenhagen, Denmark) for retting flax. This enzyme also resulted in fiber yield and properties equal to or better than fiber from water retting [37]. Later, Lyvelin (Lyven, Caen, France), a pectinase, but not pure, from *Aspergillus niger* was marketed specifically for retting of flax. Despite these positive results and developments, an enzyme retting method did not replace dew retting. While the reasons are complex, likely enzyme costs and lack of industry support prevented further development. Flaxzyme is no longer sold under this name.

### 3.2. Further Research on Enzyme Retting

In the 1990s, the United States was the largest per capita user of flax-containing textiles, but no linen or flax fiber for textiles was produced domestically. The only flax grown was for linseed. This statistic prompted the Agricultural Research Service (ARS) of the US Department of Agriculture to begin research toward developing a flax fiber program for textiles. The first goal in this initiative was to improve retting emphasizing enzymes. Results from the work in Europe, particularly with pectinases, were the basis of research. There were other considerations, however, that became apparent for this work. Canada and the US northern plains states had a thriving linseed industry, with tons of waste straw available. Linseed straw removal after harvest presented a problem for farmers. Producers wished to remove the straw from their fields soon after harvest, and the straw did not degrade readily. A small portion of this crop, estimated around 25%, was used for hammer milling and pulp for specialty papers, such as cigarette, currency, and Bible sheets. Most of the linseed straw was not used and was (and still is) burned to remove it from the fields. Further, the US textile industry was tied to cotton fiber processing and not the long-line linen processing of Europe. In fact, no specialized wet-spinning equipment required for long-line linen yarn existed in the US. The textile spinning technology was based on short staple fibers like cotton and synthetic blends. A flax tow product, which is short fiber as a by-product of long-line linen, was used in blends with cotton, but that product, too, was imported. 

So, quickly, the ARS research effort incorporated linseed stems as a source of fibers, with attempts to improve retting and processing for higher, quality in the fibers. Further, since the fibers desired were short-staple like cotton, “total fiber production,” with collection of all bast fiber regardless of length, was employed with later “cottonizing” to shorten and refine the fibers. 

The ARS flax research focused on lowering enzyme amount, finding purer and more active enzymes, and developing a protocol for enzyme retting. Earlier research indicated the value of calcium chelators for disrupting pectins in retting (see ealier). Tests with oxalic acid showed that chelators could greatly reduce the amount of enzyme needed for retting [45]. So, with following tests, the retting mixture was almost always an enzyme/chelator mix. 

Excellent field retting of flax had been noted in research experiments at Clemson University, South Carolina, for winter production of flax. From this material, the major fungi colonizing the stems were cultured and isolated in a search for more active retting enzymes. One fungus stood out from the others as the most active retter of flax [43]. This fungus, identified eventually as *Rhizopus oryzae* sb NRRL 29086, produced a potent endopolygalacturonase (EPG) and few other enzymes in the filtrate [30, 112]. This enzyme was purified and tested for its ability to separate fibers in stems and compared in mixtures with potentially complementary cell wall-degrading enzymes. In these early tests, fiber separation was judged by light microscopy and the Fried's Test, which is an *in vitro* test to judge fiber separation from stems by comparing visual images [37]. 

Results indicated that this purified EPG with oxalic acid alone was sufficient to separate flax fibers (Table 6). The other potentially complementary enzymes tested, namely, pectin methyl esterase, xylanase, and cellulases, did not improve fiber separation [30]. Data, therefore, indicated that enzymes other than EPG were not required to separate fiber from nonfiber fractions. 

Based on these results, a search for a potential commercial enzyme, with high pectinase and low cellulase activity, indicated that Viscozyme L (Novozymes North America) had similarities to SP 249 and Flaxzyme. Oxalic acid in later tests proved not to be suitable as a chelator, as a precipitate formed and remained on the fibers. While tests continued on a series of chelators of different types and under different conditions [113, 114], EDTA replaced oxalic acid because of efficiency at pH 5 to 10 and its commercial availability for the textile market. The retting test method of choice, then, was a combination of the commercial products Viscozyme L and EDTA (later using Mayoquest, a 36%–38% EDTA commercial product) for other comparisons and modifications. A series of Viscozyme/EDTA formulations, with increases in each of the enzyme and chelator, was used to ret a mature, fiber-type flax. Rather than the Fried's test, cotton fiber tests were used for strength by Stelometer [115] and fineness [116] using a modified microaire system equilibrated with a series of fineness standards fibers [117]. An estimate of the percentage of fine fiber, collected by passing retted and mechanically cleaned fiber through the Shirley Analyzer, was calculated from the starting material. The Shirley Analyzer, which is an instrument to separate and collect trash in cotton, provided a percent of fine, cleaned fiber as an additional statistic to judge quality. 

Experiments with a series of retting combinations [32] indicated that increasing Viscozyme levels increased fine fiber yield but reduced strength, regardless of chelator levels (Table 7). Increasing levels of chelator, within each enzyme series, increased fine fiber yield and resulted in finer fibers up to 18 mM. Chelator level alone did not affect strength. Fibers from this study were blended with cotton (50 : 50), spun as yarn in a miniature spinning system, and the yarn properties determined [118]. Results indicated that enzyme treatments affected yarn properties, with the highest enzyme level producing finer fibers, easier yarn construction, and better quality yarns. Assessments are difficult to compare, however, because, for example, the highest level of enzyme produced a finer but weaker fiber. Possibly, finer but weaker fiber was also less stiff and brittle and therefore more amenable for blending with cotton. Both of these characteristics are important in yarn construction. The clearest result from this work was that the miniature spinning system has value in predicting the optimal retting formulations for yarn quality.

In order to develop a new pilot plant method for enzyme retting, a spray enzyme method, or a brief (2 min) soaking, to deliver the enzyme/chelator mixture was used to reduce liquid : solid ratio compared to the former method of retting flax in submerged tests [119]. Since the cuticle/epidermis layer protected the internal stem tissues [45], methods were explored to facilitate the entry of enzymes into stems. Physically crushing stems with fluted rollers to breach this barrier improved enzyme retting over that by increased or reduced atmospheric pressures [120]. Although inhibitory, aromatic compounds had been shown to be released by chemical treatments [74], presoaking of fiber with water to remove these compounds showed no clear benefit with enzyme retting and was not included as part of the enzyme retting protocol [121]. 

The enzyme retting method, termed SER, was tested on seed- and fiber-type plants, with various levels of enzyme and chelator. Ultimately, enzyme retting would have to be integrated to a commercial cleaning system for fiber production, but such cleaning systems did not exist in the US. Accordingly, arrangements were made to produce pilot scale amounts of enzyme-retted flax for commercial processing in Europe. About 12 kg of retted flax for each formulation was commercially cleaned by Ceskomoravsky len (CML) in Humpolec, Czech Republic, using a Unified Line scutching system and La Roche cottonizing system [33]. These fibers were tested for quality parameters and then spun into blended yarns with cotton at 50 : 50 and at 10 : 90 flax : cotton amounts. Fibers were tested with modified cotton testing equipment (Table 8) and the yarn properties by commercial testing equipment and methods (Table 9). 

Results indicated that the enzyme mixtures retted both fiber- and seed-type plants (Table 8). Higher levels of enzyme reduced fiber strength but produced finer and higher amounts of fine fiber. Other than strength, fineness and fine fiber yield were better than these characteristics in dew-retted fibers. Chelator levels did not seem to vary in their impact, with 25 mM amounts equal to the 50 mM levels. The seed flax fibers were of less quality than fiber-flax fibers with similar formulations. Retesting of the fibers 30 months later showed no further loss in strength, indicating the washing step after enzyme retting was sufficient to stop further enzyme activity. Yarn properties were compared favorably between dew retted and enzyme retted at the higher level of enzyme (Table 9). Seed-flax fibers processed into blends much like the fiber-flax fibers, even the dew-retted sample. Specific areas for improvement were identified. Of concern was the loss of strength with increasing level of enzyme. 

Since retting, processing, and yarn construction are interrelated, a flax pilot plant was constructed based on the Unified Line at CML in order to have a commercial method for cleaning retted flax stems [122]. This system was developed by engineers at CML but reduced in size and with each of four parts separately positioned for research. The parts were: 9-roller calender for breaking stems, 5-roller calender for further crushing shive, scutching wheel to produce a total fiber from the stems, and an upper pinned shaker to remove loose shive and straighten fibers.

Further, a series of test protocols was developed for objective test results of fibers. Fibers were tested using cotton equipment and protocols where applicable, such as strength and elongation by Stelometer [115]. Further test standards were developed for flax, including color, fineness, and predicted shive through the Flax and Linen subcommittee of ASTM International [56, 57]. The following tests methods adopted were (1) percent of fine fiber yield produced by passing through a Shirley Analyzer, (2) tensile strength and elongation by Stelometer, (3) fineness based first on a modified cotton airflow method and later refined for a new ASTM test method [57], and (4) the percent shive in cleaned fiber using near infrared spectroscopy and chemometric models from fiber : shive combinations [54, 55, 59]. This latter method was accepted as a new ASTM test method in 2005 [57]. For certain assessments, the Fried's Test and light and scanning electron microscopy were used when appropriate. A color test method (D-6961-03) was approved as a new ASTM test method in 2003 [57]. Enzyme retting results in a lighter fiber color than that by field retting, and various enzyme retting formulations resulted in different color characteristics based on the CIELAB L∗, a∗, and B∗ values [123, 124]. These results suggest methods to tailor color properties for applications.

**Figure 8:**
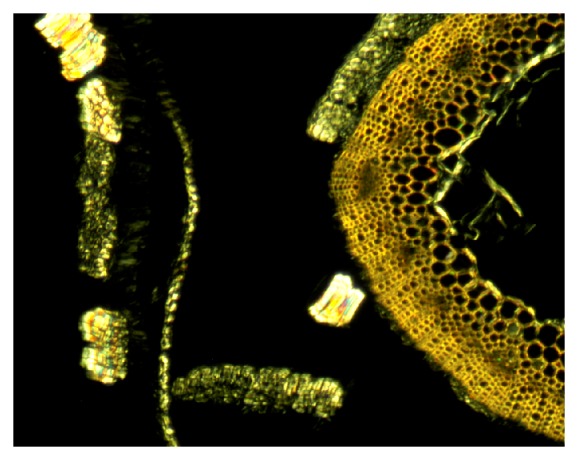
Polarized light microscopy of free-hand section of flax stem incubated with Bioprep. The birefringent fibers, in bundles, are separated from the lignified core and thin cuticle layer.

 Enzyme mixtures that included cellulases, such as Viscozyme L, weakened fibers (Figure 7), as shown by previous work (Table 8). Purer enzymes were becoming more readily available at this time, and research had showed that purified EPG alone could separate fibers from core without the other cell wall-degrading enzymes. Further, results indicated these methods worked on both fiber-type and seed-type flax cultivars, indicating that these enzymes should be applicable to linseed straw. Commercial enzyme products, developed for various applications, were tested with the intent of finding purer pectinases (i.e., low or no cellulases) that effectively retted flax without loss of fiber strength. As these further tests were being carried out, Novozymes North America, Inc. (Franklinton, NC, USA) released a commercial pectate lyase (PL) product for removing the cuticle of cotton fibers as an environmentally friendlier way of scouring cotton, which traditionally used high levels of NaOH [125, 126]. BioPrep 3000L is a liquid commercial PL produced by multiplying the native gene for alkaline PL in *Bacillus lichniformis*, placing the genes back into the bacterium and allowing expression of these genes for high levels of enzyme production. Bioprep has a reported activity of 3,000 alkaline pectinase standard units (APSU)/g. We used a product marketed under the trade name Dextrol Bioscour 3000 (Dexter Chemical LLC, Bronx, NY) [35], which was shown to separate the fibers (Figure 8). 

At this time, other enzymes were developed and applied especially for enzyme retting [127, 128]. Texazym BFE and Texazym DLG are propriety names for enzymes from Inotex Ltd., Dvűr Královi, Czech Republic, specifically mentioned for use in field retting. Other commercially produced enzymes for various applications of degradation of plant materials were selected. Fibers produced by various enzyme retting formulations were assessed through use of the fiber processing pilot plant and objective test methods [34]. All the enzymes were tested using suppliers' recommendations for optimal activity.

 The Fried's Test suggested initial levels and times for effective flax retting of these various enzymes and formulations [34]. Texazym BFE effectively separated fiber from core at 2%, 5%, and 10% levels after 24 h; only 10% BFE retted flax at 7 h. The addition of EDTA (18 mM concentration) improved retting, showing effective fiber separation at 7 h for 5% BFE. EDTA at 18 mM concentrations improved retting of all enzymes except DLG, which alone was ineffective in fiber separation by this method.

 The effect of retting was further evaluated using BFE, DLG, Multifect Pectinase FE, and Bioprep 3000 L in several modifications of formulas and retting conditions [34]. While 1% BFE was effective at 24 h, the addition of EDTA facilitated enzyme retting with all levels of this enzyme. The 2% level appeared to be effective enough to warrant further study, and temperatures in the 50 to 60°C range were more effective than lower temperatures. Incubation of stems with DLG at 5%, even with EDTA,did not result in fiber separation by this test. Multifect Pectinex FE was effective at 0.2% with EDTA, but not without the chelator; lower levels were less effective than other enzymes even with EDTA. Addition of DLG as high as 0.5% did not improve fiber separation efficiency of 1% BFE plus EDTA by the Fried's Test. Similarly, addition of xylanase, to treat animal feed and reported low in cellulase activity, included up to 0.15% with Multifect FE plus EDTA did not improve fiber separation. Bioprep 0.05% at pHs 8 and 9 and with chelators effectively separated fibers from the core. 

 Many of the enzyme mixtures tested contain multiple types of enzymes active against plant cell walls, including cellulases. A companion study was carried out to test the activities of several commercial polygalacturonases from various sources [129]. Tests of enzyme activities and flax fiber properties, including strength, indicated different cellulase activities within these products that affected fiber properties. Microscopic analysis and incubation of commercial flax fibers with these enzymes over several days shows clear signs of fiber degradation by many. Texazym BFE and Bioprep, however, resulted in slight to no fiber destruction. Bioprep is listed as an alkaline pectate lyase, while the optimal conditions for activity of Texazym, which is not identified as to type of pectinase, is similar to that for Bioprep. In contrast, Texazym DLG and Sigma cellulase were very destructive to flax fibers. It should be noted that most of these commercial enzymes are not marketed for flax retting. Their use, for example, Texazym DLG, however, could modify fiber and yarn properties as will be discussed later. 

Stems retted with various enzyme formulations by the SER method and with fiber processed through the pilot plant are shown in Table 10. Fine fiber yield was highest for Texazym BFE and Bioprep formulations but not significantly different from Viscozyme plus Mayoquest. All enzyme-retted and Shirley-cleaned samples were cleaner than unretted fiber, and differences were not large among the enzyme treatments. Differences, however, occurred in strength and fineness among retting formulations.

Further evaluation of the use of these enzymes for retting included the following: the amount of formulation uptake during brief (i.e., 2 min) soaking, fine fiber yield, and cleanliness [35]. Uptake of the amount of various formulations of Bioprep was similar and about 300 mL (ranging from 272 to 408 mL) for 150 g initial fiber, giving a liquid-to-fiber ratio of 2–2.7 to 1. 

Additional assessments tests were made on linseed varieties grown under commercial-type conditions in North Dakota [35]. Results showed that all retting enzymes were more efficient with chelating agents, particularly EDTA. EDTA has substantial Ca^2+^  binding activity even at pH 5 [114], providing a positive effect of EDTA at low pHs, which is optimal for some enzymes such as EPG and Viscozyme. The binding capacity of EDTA for Ca^2+^  is, however, considerably greater at alkaline pH [114], and the use of EDTA at a higher pH should be more efficient in separating fiber from core. It is well known, however, that PL requires Ca^2+^  for activity [77]. The suggested method for cotton scouring with Bioprep is to first apply the enzyme and later apply the chelator (S. Salmon, Novozymes, personal communication). 

The enzyme retting methods developed earlier indicated that Viscozyme could ret linseed varieties of flax but reduced fiber strength. Tests were conducted on the efficiency of Bioprep and Viscozyme to ret two linseed varieties, and fiber properties were determined. Hermes and Omega were grown to full seed maturity under production conditions in North Dakota. Stems were enzyme retted using formulations with Bioprep or Viscozyme in side-by-side tests (Table 11). The Omega sample had rain prior to baling, and substantial weathering had occurred as indicated by darkening of the straw. Hermes, in contrast, was light and showed no effects of weathering prior to enzyme retting. Bioprep effectively retted both cultivars and resulted in higher fiber yield and fiber strength than Viscozyme. Hermes was finer after retting with Viscozyme plus chelator (Table 11). In this test, the chelator was used subsequent to the soaking with Bioprep but with the Viscozyme in a single solution. 

Tests for incubation times with Bioprep, level of Bioprep (without chelator), and levels and incubation times of chelator were further assessed [35]. Based on fine fiber yield and percentage shive content, incubation with Bioprep for 1 h followed by incubation with 18 mM EDTA for 24 h was equal or better than other conditions. Retting effectiveness, however, improved with increased amounts of Bioprep up to 0.5%, which was the highest level tested in this experiment and suggested that further increases in enzyme level may improve fiber separation. Furthermore, scanning electron microscopy of retted fibers indicated that Bioprep levels of 5% appeared to remove more contaminants than 0.1%. 

Based on earlier results and general recommendations for bioscouring cotton with Bioprep (personal communication, S. Salmon, Novozymes North America, Inc.), a series of evaluations was carried out to optimize the use of Bioprep and EDTA for retting flax (Table 12). Linseed variety Hermes was selected for these tests. The recommendation for bioscouring cotton was to treat with Bioprep about 15 min prior to adding chelators (S. Salmon, personal communication). The use of Mayoquest 200 to supply EDTA as chelator at 18 mM concentration, which had been determined from use with Viscozyme, appeared to work adequately with Bioprep. To further optimize the formulation and method for enzyme retting, Hermes was retted with a range of Bioprep levels from 0.1% to 5% and followed by chelator or combined with chelator in the formulation. The higher levels of “fiber” with the lower enzyme levels arise from fiber plus shive in varying amounts, as shown by predicted shive amounts. For Shirley-cleaned fine fiber, Bioprep at 1.0% to 5.0% followed by chelator produced the highest fiber yields and the lowest shive contents, ranging from 1.5% to 2.3%. Bioprep at 5% did not produce higher yields or cleaner fibers than 1% or 2% levels. Shirley-cleaned fibers do not represent all the fibers that could be extracted in commercial, cottonizing systems. Therefore, fiber yields from a single pass through the Shirley Analyzer were used only to rank enzyme formulations. 

Fiber strength was maintained at all levels of Bioprep (Table 12), showing a significantly greater strength than for fibers retted with Viscozyme (Table 11). Therefore, a major objective of enzyme retting with increased fiber strength was reached with pectate lyase followed by EDTA. The commercial enzymes used in the present study represented a mixture of polysaccharidases, for example, cellulases and hemicellulases in some, as well as different types of pectinases. Viscozyme, or EPG, and pectate lyase were effective in attacking pectin and retting flax, but the two enzymes have different optimal conditions for activity and different modes of action. 

Advances for flax fiber processing could occur with specific enzymes and systems. Polygalacturonase (PG) and pectate lyase (PL) are both depolymerizing enzymes for pectin but work in different ways and under different conditions. PG is reported to catalyze random hydrolysis of *α*-1,4 polygalacturonic acid, and PL carries out a nonhydrolytic breakdown of pectates and pectinates by a trans-elimination split of the pectic polymer [77]. PL is activated by Ca^2+^ and usually is active at higher pHs (e.g., 8–10) and temperatures (55–60°C) than PG. Research has been carried out to correlate fiber separation with the degradation of different “pectins,” that is, various functional groups and linkages, using a series of commercial PGs and PL with low xylanase and cellulase activities [130]. Retting efficiency was highly correlated (correlation coefficient of 0.99) with sparsely esterified pectin, but correlations between retting and activities against other pectins were low. Since Ca^2+^ binds acidic groups of pectin molecules and various types of pectin are in different regions of the bast [66, 81], these data further reveal a coordinated mechanism for degradation of nonesterified flax pectins with chelators and pectinases. Further, these and other spectroscopic data [131] suggest that the pectins in the middle lamella and those binding the cuticle/epidermis to fiber bundles in flax stems are targeted by this mechanism. Towards a more cost-effective enzyme retting system along these lines, other work [132] indicated that weak acid with enzymes was effective in separating bast fibers with reduced enzyme levels, likely by removing the Ca^2+^ in pectin.

Work with commercial PL has indeed shown efficiency in separating bast fiber from stems (Figure 8, Tables 10–12). Bioprep levels around 2% with 18 mM EDTA were optimal with the flax samples used and conditions tested (Table 12). Fiber yield, fineness, and cleanliness were not improved with higher Bioprep levels. Sequential treatment of Bioprep followed by EDTA was the most effective for retting, but combining both enzyme and EDTA also retted flax. The procedure most effective for producing fine, clean fiber was as follows: (a) saturate crimped flax stems with Bioprep at 2%, (b) incubate for 1 h at 55°C, (c) without washing, resoak with 18 mM EDTA at pH 12, (d) continue incubation at 55°C for about 24 h total time, and (e) wash and dry fiber in preparation for mechanical cleaning. 

Other work has shown the potential of alkaline pectinases such as PL to ret flax and the bast plant ramie [127, 133]. Bioprep-treated fiber was not tested in the miniature spinning system or for biocomposites to this author's knowledge. An engineered pectate lyase from *Xanthomonas campestris, *however, was developed and used at 37°C and pH 8.5 to “bioscour” commercially grown and decorticated linseed flax [134–136]. Matched PL-treated and untreated fibers were then used in manufactured biocomposites. PL-treated fibers were cleaner and finer than untreated fibers. In some biocomposites, PL-treated fibers performed better than similar but untreated fibers [134]. Other linseed straw samples, which had been left in the field for a few weeks and then decorticated and treated with *X. campestris* PL for various times up to 46 hr followed by chelator, did not result in improved biocomposites [135]. Still further tests of commercially decorticated linseed straw showed that PL-retted fibers, although finer and cleaner than untreated ones, performed better in tensile strength tests but not in interfacial shear strength tests [136]. Further assessment is required to optimize use of Bioprep and other pectate lyases as a retting enzyme for flax. It is clear that many factors influence the successful production and processing of fibers for textiles and biocomposites. Further, while weak cellulases, such as those in Viscozyme, reduced fiber strength, the resulting finer fibers often spun better than fibers produced by lower Viscozyme levels in blends with cotton. Further assessment is required on fiber characteristics for specific applications (e.g., blended textiles or biocomposites), as well as the economics of enzyme retting. It is clear, however, that pectate lyases can separate fiber of nonfiber components and retain fiber strength. The designed purpose of Bioprep as a cotton scouring agent, which acts by removing the cotton fiber cuticle, has shown to be effective in several large tests [125, 126]. 

The ARS research on enzyme retting of flax had ended by 2012, with the retirement of key individuals and closing of the USDA pilot plant. Research and development continues for retting and other fiber applications with enzymes [127, 134]. Inotex (Dvur Kralove n.L., Czech Republic) has developed enzymes to assist with field retting [128], particularly towards producing consistent fibers in varying weather conditions and including use of oilseed straw (J. Marek, Inotex, Czech Republic, personal communication). 

Genetics for plant modification of flax to improve fiber properties for linen and biocomposites are active areas of research. Related to the idea of improved retting, some research is focused on genetically modifying flax for improved fiber extraction from linseed stems (Michael Deyholos, University of Alberta, Canada, personal communication). One goal of another program, FIBRAGEN, is to identify genetic markers, including those determining anatomy and physical properties, for expanding flax markets in textiles and biocomposites (Jörg Müssig, Hochschule Bremen, University of Applied Sciences, Department for Biometrics, personal communication). Advances in plant modification coupled with knowledge of specific action of enzyme systems for extracting fiber bode well for new systems to economically extract fibers of high and consistent quality and for directed purposes. 

### 3.3. Enzymes for Postharvest Treatment of Flax Fiber and Yarns

Perhaps one of the most effective uses of enzymes may be in postharvest treatments of flax fibers to impart specific properties. Fiber-modifying enzymes are marketed for a variety of purposes, including enrichment of dew retting, repair of poor quality flax (such as underretted material), cottonization of bast fiber tows for textiles or biocomposites, tailored fiber length, and processing of rovings to reduce noncellulosic content [128]. The use of enzymes pertinent to field retting includes spray applications after stalk pulling to minimize the effect of inclimate weather and to better utilize linseed stalks in Europe (J. Marek, Inotex, Czech Republic, personal communication). Weak cellulases may have applications where precision in limited attack on cellulose may be beneficial, such as for cottonization or shortening of fiber. To this purpose, laboratory tests of flax pulp treated with commercial pectinases and cellulases showed improved characteristics of hand sheets compared to those prepared by traditional (nonenzymatic) methods [137]. For pulping, the breakdown of the fiber bundles by pectinases and the shortening of the cellulosic fibers by attack of cellulases at the fibernodes improved some paper properties in laboratory studies. The nature of flax fibers, that is, the lack of limiting lignin in bundles and presence of susceptible fibernodes, provided opportunities for use of these enzymes not possible in highly lignified, woody sources of pulp. The authors further suggested that a more precise attack by specific enzymes may provide additional attributes in the pulp. 

Research with an atomized enzyme delivery system showed that endoglucanases could be effectively delivered in small amounts onto field-retted fibers, likely resulting in attack at the fibernodes to reduce fiber length, strength, and elongation [138]. Application of the atomized method with endoglucanase and extended to other enzymes [36] modified the properties in flax fiber and in flax/cotton blended (50/50) yarns (Table 13). These enzymes were used as supplied, and such mixtures usually have multiple enzyme profiles against fibers [34, 109, 129]. While further work is needed to assess specific activities, data suggest that all enzyme types were active in atomization, and various properties could be modified. For example, lipase and arabinase improved certain yarn properties, such as increased strength and elongation and reduced neps and thick and thin places. Results further suggest that precise enzyme activities could tailor fiber and yarn properties.

### 3.4. Enzyme Retting of Other Bast Plants

This paper has focused on flax structure and composition, with potential for enzyme retting. Emphasis has been placed on the nature of the flax bast fiber and bundle, specifically the lack of high levels of lignin, the binding of Ca^2+^ in pectin molecules in the cuticle, and the presence of the more susceptible fibernodes and kink bands within the fibers and bundles. Pectinases, either polygalacturonases or pectate lyases, alone are able to separate fibers from cuticle and core. Flax is just one of many bast plants that are economically important for myriad uses throughout the world. Would the same enzyme work for other bast plants as for flax? Research suggested that bast fibers with other characteristics may require other types of enzymes. Ramie, which is a nonlignified, cellulose-rich bast fiber-like flax (unpublished data), has been retted with pectin lyase [133]. Enzyme retting of hemp, which is more heavily lignified than flax, showed some success, but different enzymes or protocols from those with flax were needed [139]. Kenaf is highly lignified in the secondary walls and middle lamella of the bast fibers and bundles and has been retted by chemical means [140, 141]. Use of a commercial enzyme, having cellulase and xylanase activities, with chelators and a crimping pretreatment separated the bast tissue to fiber bundles [139]. This process only produced coarse fiber bundles, and a delignifying process seems to be required for effective retting of kenaf. To this end, enzymes from noncellulolytic, lignin-degrading white rot fungi to remove aromatics and leave cellulose [142, 143] may find applications. Successful, cost-effective, and commercial technologies will have requirements such as the following: selected flax material, enzyme formulations and conditions to tailor fibers with specific properties, integrated cleaning procedures, objective assessment methods to assure high and consistent quality, and directed applications. 

## 4. Summary and Conclusions

Flax has had a long and illustrious impact on human development for millennia. The long, fine, and strong bast fibers provide apparel and other textiles, and other varieties of flax provide linseed and its oil. The textile industry that once flourished in western Europe has declined, but the desire for flax and linen is still strong. Quality fibers are still marketed in Europe, and China and other regions desire more quality flax fibers for products. Even the paper and pulp industries desire cleaner fibers to reduce the amount of chemicals required for delignification. Biocomposites and nonwoven materials are predominant areas of interest, with the automotive industry continuing to focus on natural fiber composites. In particular, biocomposites are sought for weight and cost savings, improved structural properties, processing benefits, and design flexibility and ease. Compared to glass, flax fibers are lower in cost, lower in density, biodegradable, and similar in elongation at break; tensile strength is lower for flax. Woven flax fibers as insets with resins particularly provide good strength and rigidity in composites. Substantial savings in energy costs are possible with natural fiber mats, which reportedly require about 80% less energy than those made with glass. Flax fiber provides a low cost alternative for glass fiber in reinforced composites. The replacement of glass fibers with flax for this application, even with its important advantages, is nonetheless a considerable challenge. 

Consistency in supply and in fiber characteristics is required for flax fiber to expand further into markets, especially higher value-added products. Reportedly, the best flax fiber is still produced in western Europe, where climate and grower experience provide quality fibers for textiles. The drive for fibers in biocomposites and other industries has focused on getting flax fiber from nontraditional linen plants, namely, linseed straw. Field retting, which is the primary method of flax production, is problematic in that the fibers are often poor and inconsistent in quality. Climate is a major factor in quality, and outside western Europe, the major areas of flax production are often in harsh climates for field retting. It is in regard to all these factors that research has focused on other methods, including enzyme retting, to improve retting and thereby fiber processing and quality.

Replacement methods for field retting have been sought for a long time, but currently, there are no such methods used commercially. Enzyme retting has been researched for several years and is still undergoing development. There have been positive results, and there is considerable interest in postretting and treatment of roving and yarn to improve their properties. New developments in enzyme production by commercial companies have provided purer and more active pectinases that have promise in enzyme retting. Our work examined several commercial enzymes for retting and focused on the endopolygalacturonase-rich, mixed product Viscozyme plus EDTA and the purer alkaline pectate lyase product Bioprep followed by a commercial EDTA chelator. Protocols were developed on enzyme concentrations and conditions to separate fibers, which were then cleaned in a fiber processing pilot plant and characterized by objective test methods. While Bioprep-retted fibers had good properties of fineness, strength, and cleanliness, tests in textiles or composites have not been carried out. The vast amount of research on enzyme retting indicates that pectinases without the need of complementary enzymes are effective in separating fiber from flax straw, even linseed straw. The inclusion of a chelator, such as EDTA, greatly reduces the amount of enzyme required and is particularly effective in separating fibers from the cuticle-epidermis layer in linseed straw. Fiber properties can be tailored with the use of specific enzymes.

Improving the quality and consistency of fiber from the huge biomass resource of linseed straw has great potential in addressing needs of myriad industries, even that of cottonized flax fiber for textiles. The degree of processing for fiber cleanliness will depend upon the end product desired, and for some products, the requirements of cleanliness and processing costs are considerably less than for linen fabrics. The desire for quality apparel, however, continues to be important in flax and linen products, and improved processing methods should not overlook this important and historical industry. To this end, the Crailar process reportedly uses enzymes in a proprietary process to produce soft, fine flax fibers for blending with cotton in an agreement with the Hanes clothing industry. One of the most important uses of enzyme might be in tailoring specific properties in postharvested materials to improve low-quality fibers.

Fibers and seeds are two historical products of flax with traditional and continuing economic importance around the world. It is likely, however, that the usefulness of flax will not be limited to just fiber and seed, as physical, chemical, and biotechnical methods uncover more products. Production of flax fibers by enzymatic or other means of retting, followed by mechanical processing, generates bast fibers for many industrial needs and massive amounts of by-product wastes. This waste material, consisting of cuticle, shive, and fiber fragments, is already paid for and is localized at the processing plant. The potential for coproducts from processing flax fiber is huge. Mention has been made of lipids (sterols, policosanol-type lipids, and waxes) from the cuticle in dust and of activated carbons and extracted aromatics and sugars from the shive. Currently, there is a burgeoning interest in microcrystalline cellulose from plants and their potential for value-added products ranging from biocomposites for medical devices to solidified liquid crystals. Considerable work is still needed to overcome substantial problems and directed applications towards reaching the huge potential for cellulose nanocomposites. The highly crystalline and oriented nature of cellulose in flax fibers warrants consideration for its properties in nanotechnology. The source, chemistry, structure, and crystalline nature of the native flax bast fibers, particularly in regard to the response to specific enzymes, may offer a contribution to this growing area of research and technology. 

Indeed, linen most useful, *Linum usitatissimum*, is poised to continue to expand as a supplier of useful products to mankind throughout the world.

## Figures and Tables

**Table 1:** Chemical composition (mg/g) of the cuticle/epidermis layer of flax stems.

Component	Cultivar and harvest time^1^			
	Laura (mature)	Omega (mature)	Ariane (fiber)	Ariane (mature)
Total aromatics	5.8	7.6	2.2	3.0
Total wax	63.6	36.4	37.7	39.4
Total cutin	84.1	35.1	80.8	43.7
Total sterols	1.7	0.9	0.3	0.3
*Selected lipids *				
C-16 fatty acid	16.9 ± 2.8	8.8 ± 0.7	14.5 ± 3.9	9.7 ± 0.5
C-18 fatty acid	10.8 ± 0.3	6.1 ± 0.4	6.7 ± 0.5	5.3 ± 0.3
C-28 alcohol	18.9 ± 2.4	12.6 ± 0.5	6.0 ± 2.6	7.9 ± 1.0
8,(9),16-dihyroxy C-16 fatty acid (mixture)	73.7 ± 15.0	31.0 ± 2.4	71.9 ± 2.9	38.4 ± 1.8
Percentage of bast	18.3	24.1	13.9	21.0

^1^Laura and Ariane are fiber types. Omega is a seed type. Mature refers to harvest time at full seed maturity, while fiber refers to harvest for optimal fiber quality. Data adapted from [23].

**Table 2:** Carbohydrate composition of flax core cells.

Plant fraction^1^	Carbohydrate level (mg/g)						
	Uronic acid	Rhamnose	Arabinose	Xylose	Mannose	Galactose	Glucose
Core (N)	0.3 ± 0	17.3 ± 3.1	9.4 ± 2.5	166.5 ± 0.7	13.1 ± 0	8.8 ± 1.6	270.0 ± 11.3
Core (A)	0.3 ± 0.2	10.1 ± 4.1	7.2 ± 0.3	136.5 ± 3.5	14.7 ± 4.0	9.8 ± 2.2	277.0 ± 26.9
Core (A) *d *	0.2 ± 0	6.4 ± 0.2	3.8 ± 0	132.5 ± 17.7	9.4 ± 3.9	5.9 ± 0	247.0 ± 44.5

^1^The core and fiber materials were hand separated from plant stems. (N) is the cultivar Nastasja, which was grown in South Carolina. (A) is the cultivar Ariane grown commercially and supplied by Van de Bilt Zaden b.v. Sluiskil, The Netherlands. *d:* dew-retted by Van de Bilt.

Data adapted from [24].

**Table 3:** Aromatic composition of flax core.

Plant fraction	Aromatic content (mg/g)			
	Ferulic acid	Guaiacyl units	Syringyl units	Total
Natasja whole	0.4 ± 0	5.7 ± 2.9	3.9 ± 2.1	10.4 ± 4.9
Natasja lower	0.4 ± 0	7.8 ± 1.7	5.7 ± 0.8	14.4 ± 1.8
Ariane unretted	0.6 ± 0	9.5 ± 1.1	7.8 ± 3.3	17.9 ± 2.1
Ariane dew-retted	0.7 ± 0.1	8.4 ± 1.3	4.0 ± 2.8	13.0 ± 3.7

Data adapted from [24].

**Table 4:** Carbohydrate composition of flax fibers.

Source^1^	Carbohydrate concentration (mg/g)						
	Uronic acid	Rhamnose	Arabinose	Xylose	Mannose	Galactose	Glucose
Fiber (A)	0.2 ± 0	9.7 ± 0.6	15.5 ± 0.6	15.9 ± 1.7	30.7 ± 0.9	32.4 ± 0.4	434.0 ± 18.3
Fiber (A) *d *	0.1 ± 0	7.6 ± 2.9	5.5 ± 1.2	7.0 ± 0.2	39.1 ± 2.5	34.9 ± 0.9	649.5 ± 38.8
Fiber (A) *f *	0.1 ± 0	8.4 ± 0.4	5.5 ± 0.1	6.6 ± 0.2	37.3 ± 1.3	41.3 ± 1.5	699.0 ± 5.7
Fiber (A) *u *	0.1 ± 0	6.6 ± 1.5	5.3 ± 0.8	7.6 ± 0.1	33.4 ± 0.6	27.7 ± 0.8	602.0 ± 24.0
Fiber (A) *e *	0.1 ± 0	14.8 ± 0.4	11.4 ± 1.1	10.7 ± 0.8	31.5 ± 4.0	28.3 ± 2.1	498.0 ± 40.0
Fiber (L) *l *	0.3 ± 0	14.8 ± 5.8	9.6 ± 4.9	17.2 ± 9.1	31.8 ± 1.6	30.2 ± 0.7	563.0 ± 2.8
Fiber (L) *h *	0.3 ± 0	11.9 ± 1.3	10.9 ± 2.9	12.4 ± 1.2	45.0 ± 0.1	36.9 ± 2.3	719.5 ± 4.9
Fiber (B) *w *	ND^2^	6.7 ± 0.6	4.7 ± 0.2	12.6 ± 1.6	45.6 ± 4.6	35.3 ± 3.6	668.9 ± 31.3
Fiber (M) *d *	ND	6.9 ± 1.3	6.9 ± 1.3	10.7 ± 1.6	44.6 ± 8.8	38.9 ± 4.8	661.8 ± 50.3

^1^The core and fiber materials were hand separated from plant stems, with the fibers cleaned by various means. (A) is cultivar Ariane grown commercially and supplied by Van de Bilt Zaden b.v., Sluiskil, Holland. (L) is commercial fiber from an unknown cultivar supplied by Linificio and Canapificio Nazionale (Bergamo, Italy). (B) is from 5 water-retted Belgium samples. (M) is from 6 dew-retted European harvests. Lower case letters following refer to the following treatments: *d:* dew-(field-)-retted by Van de Bilt, *f*: retted by Flaxzyme (Novo Nordisk, Denmark, supplied by H.S.S. Sharma), *u*: retted by Ultrazym (Novo Nordisk, Denmark), and *e*: retted by EPM (Genencor International, USA). Fiber (L) *l* is a low quality commercial grade of fiber. Fiber (L) *h* is a high quality commercial grade of fiber.

^2^Not determined. Data adapted from [25–27].

**Table 5:** Aromatic and lipid composition of flax fibers (mg/g).

Source	Total aromatics	Cutin^1^	Waxes
Natasja whole	2.9 ± 0.4	ND^5^	ND
Natasja lower	3.8 ± 0	ND	ND
Ariane unretted	7.2 ± 1.9	ND	ND
Ariane dew-retted	Trace	ND	ND
Ariane mature (man)^2^	1.1	0.3	1.6
Ariane early (man)^2^	0.8	0	0.8
Laura (man)^2^	0.5	0.1	1.3
Omega (man)^2^	2.1	0.1	2.8
Fiber (B) *w* ^3^	0.9 ± 0.2	8.8 ± 5.2	13.5 ± 5.0
Fiber (M) *d* ^3^	0.7 ± 0.1	4.6 ± 1.7	5.9 ± 1.5
Ariane mature er2^4^	ND	3.7 ± 0.2	3.7 ± 0.2
Ariane early er2^4^	0.8 ± 0.3	3.9 ± 1.1	3.0 ± 0.8
Omega er2^4^	ND	8.8 ± 2.0	8.1 ± 1.4
ND seed flax er2^4^	1.7 ± 0.6	10.9 ± 0.8	6.9 ± 1.7

^1^Cutin represented by analysis for 8,16-dihydroxyhexadecanoic acid.

^2^Fibers manually separated without any evidence of nonfibrous material.

^3^See Table 4 for description. ^4^Enzyme-retted 2 X.

^5^Not determined.

Data adapted from [23–25, 28].

**Table 6:** Fiber separation and strength by purified enzymes.

Enzyme formulation^1^	Fried test score	Strength
Endopolygalacturonase (EPG)	2.3 ± 0.4	29.7 ± 3.3
EPG + xylanase (xyl)	2.4 ± 0.5	29.8 ± 5.3
EPG + endoglucanase (EG)	2.3 ± 0.5	26.9 ± 3.7
EPG + Xyl + EG	2.0 ± 0	22.3 ± 1.6
Xyl	1.1 ± 0.2	ND
EG	1.4 ± 0.5	ND
EPG + Xyl +EG + Celluclast	2.4 ± 0.2	ND

^1^EPG is endopolygalacturonase purified from *Rhizopus oryzae* sb (NRRL 29086) used at 18.1 U/mL to equal pectinase activity in SP 240; Xyl is a cloned xylanase from *Thermatoga maritima* [29]; EG is endoglucanase Novozymes SP 613; Celluclast is a Novozymes cellulase from *Trichoderma reesei* ATCC26921. All enzyme mixtures contained 50 mmol oxalic acid as chelator.

Data from [30].

**Table 7:** Effect of enzyme and chelator amounts on properties of mature Ariane flax stems.

Viscozyme/Mayoquest 200 (%)^a^	Fine fiber yield^b^ (%)	Strength^c^ (g/tex)	Fineness^d^ (airflow)
0/0	4.3 ± 1.7^f^	26.9 ± 0.8^a^	8.0 ± 0^a^
0.05/0.4	5.4 ± 2.2^ef^	24.0 ± 1.4^abc^	7.7 ± 0.1^abc^
0.05/0.7	7.0 ± 1.8^bcde^	23.9 ± 5.5^abc^	7.9 ± 0^ab^
0.05/1.8	8.5 ± 0.6^abc^	24.6 ± 2.3^ab^	7.7 ± 0.1^abc^
0.1/0.4	6.2 ± 1.3^def^	20.3 ± 2.5^bcd^	7.6 ± 0.1^abc^
0.1/0.7	7.3 ± 1.8^bcde^	17.9 ± 2.3^de^	7.6 ± 0.1^abc^
0.1/1.8	7.9 ± 1.2^abcd^	20.3 ± 1.8^bcd^	7.1 ± 0^cde^
0.2/0.4	6.7 ± 0.9^cde^	18.1 ± 0.6^de^	7.4 ± 0.1^bcde^
0.2/0.7	7.9 ± 1.3^abcd^	17.6 ± 0^de^	7.0 ± 0.5^de^
0.2/1.8	8.9 ± 2.1^ab^	17.7 ± 1.4^de^	6.9 ± 0.4^e^
0.3/0.4	5.5 ± 0.9^ef^	15.3 ± 0.5^e^	7.5 ± 0.7^abcd^
0.3/0.7	7.3 ± 1.1^bcde^	18.1 ± 1.3^de^	6.9 ± 0.1^e^
0.3/1.8	9.8 ± 0.8^a^	19.5 ± 0.7^cde^	6.9 ± 0^e^

^a^Viscozyme L (Novozymes) added as percentage of product as supplied. EDTA was supplied as Mayoquest 200 with 38% EDTA and used to enzyme ret mature Ariane stems.

^b^Enzyme-retted straw passed through hand-card 2X and passed 1X through a Shirley Analyzer.

^c^Average and standard deviation of 2 replicates of Shirley-cleaned fiber, with each replicate an average of 6 tests by Stelometer (force at break divided by weight by standard cotton testing).

^d^Average and standard deviation of 2 replicates of Shirley-cleaned fiber, with each replicate an average of 2 tests by air flow using approximately 5 g based and IFC flax fiber standards for fineness (similar to methods used for cotton fineness).

^a,b,c,d,e,f^Within columns, values with different lower case letters differ at *P* < 0.05.

Methods in [31]. Data from [32].

**Table 8:** Properties of enzyme-retted and commercially cleaned and cottonized flax fiber.

Fiber sample^a^	Viscozyme/EDTA (%/mM)	Fineness (air flow)	Strength (g tex^−1^)^b^	Fine fiber yield (%)
Seed flax straw	0.05/50	5.9 ± 0.1^b,c^	25.9 ± 2.9^b,c^	25.3 ± 1.0^e,f^
		6.1 ± 0.1	24.7 ± 1.9	
Seed flax straw	0.05/25	6.0 ± 0.2^b^	19.6 ± 1.1^d,e^	23.6 ± 1.0^f^
		6.1 ± 0.1	25.3 ± 3.0∗	
Ariane (early)	0.05/50	5.8 ± 0.1^c,d^	24.0 ± 2.0^c^	30.7 ± 8.8^d,e^
		5.5 ± 0.1	25.0 ± 4.5	
Ariane (early)	0.05/25	5.7 ± 0.1^d^	20.9 ± 1.3^d^	37.9 ± 0.2^b,c^
		6.1 ± 0.1	25.3 ± 3.0∗	
Ariane (early)	0.3/50	3.9 ± 0.1^g^	13.0 ± 1.3^g^	61.4 ± 0.7^a^
		4.1 ± 0	16.3 ± 1.9∗	
Ariane (early)	0.3/25	4.6 ± 0.1^f^	15.8 ± 1.8^f^	58.7 ± 1.1^a^
		4.5 ± 0.2	15.6 ± 2.1	
Ariane (early)	dew-retted	5.3 ± 0.1^e^	36.2 ± 2.3^a^	43.0 ± 1.1^b^
		5.3 ± 0.2	32.5 ± 2.2∗	
Ariane (late)	0.05/50	6.7 ± 0.1^a^	26.8 ± 3.4^b^	32.3 ± 0.3^c,d^
		7.1 ± 0.1	28.6 ± 3.7	

^a^Seed flax straw was from North Dakota, USA and grown in 1999. Ariane was either grown optimally as a winter crop in South Carolina, USA, 1999, for fiber (early) or to maturity for seed (late). Flax stems were enzyme retted by soaking for 2 min and washing with water. The fiber was cleaned in a commercial system in Humpolec, Czech Republic through the Unified Line followed by cottonizing by the La Roche system.

^b^The first number in each column is for samples tested in August, 1999 (2–4 months after retting), and the second number is for the same samples tested April, 2002 (30 month later).

^a−g^Within columns and for samples tested August, 1999, values with different lower case letters differ, *P* < 0.05.

∗Within columns and for a particular sample, values for two test dates differ, *P* < 0.05, using the *t* test.

Data from [33].

**Table 9:** Properties of yarns made with enzyme-retted flax fibers and cotton.

Fiber sample^a^	Viscozyme/EDTA (%/mM)	Single end strength (g/tex)^b^	Mass evenness (CV)^b^	Nep imperfections/1000 yards^ b^
Seed flax straw	0.05/50	10.6 ± 2.0	35.9	2659
		14.7 ± 3.1	25.5	628
Seed flax straw	0.05/25	8.7 ± 2.2	38.7	3658
		13.0 ± 3.2	27.1	647
Ariane (early)	0.05/50	11.2 ± 2.0	38.3	3373
		13.8 ± 3.3	28.9	736
Ariane (early)	0.05/25	9.3 ± 1.8	39.9	3250
		13.9 ± 4.0	25.2	597
Ariane (early)	0.3/50	9.7 ± 1.6	36.7	2961
		11.4 ± 3.1	24.6	572
Ariane (early)	0.3/25	10.0 ± 1.7	35.1	2312
		13.9 ± 2.5	26.1	571
Ariane (early)	Dew-retted	9.8 ± 1.7	38.7	2811
		13.7 ± 2.4	24.6	555
Ariane (late)	0.05/50	9.3 ± 1.9	43.5	3205
		14.3 ± 2.3	25.2	665
Upland cotton	NA	17.4 ± 1.8	19.4	461

^a^Seed flax straw was grown in North Dakota, USA in 1999. Ariane was grown optimally as a winter crop in South Carolina, USA, 1999, for fiber (early) or to maturity for seed (late).

^b^The top value in each column is a 50/50 blend of flax and cotton. The bottom number in each column is a 90/10 cotton/flax blended yarn. The flax was commercially cleaned through the Unified Line and LaRoche cottonization systems.

Data adapted from [33].

**Table 10:** Properties of mature Ariane flax fiber enzyme retted with various commercial products.

Retting formulation^1^	Fine fiber yield (%)^2^	Strength (g/tex)^3^	Fineness (SSI)^4^	Predicted shive (%)^5^
2.0% Texazym BFE + M	7.0 ± 1.2^a^	36.7 ± 1.5^ab^	4.7 ± 0.1^ab^	2.7 ± 0.8^bc^
5.0% Texazym BFE	7.2 ± 0.6^a^	34.6 ± 2.0^b^	4.3 ± 0.3^bcd^	3.3 ± 0.3^b^
0.2% Multifect Pectinase + M	2.2 ± 0.2^c^	17.8 ± 2.2^d^	4.1 ± 0.1^bcde^	1.2 ± 0.7^c^
0.1% Bioprep	6.0 ± 1.1^ab^	33.2 ± 2.4^bc^	3.8 ± 0.1^cde^	4.1 ± 0.2^b^
0.1% Bioprep + M	7.7 ± 1.4^a^	34.9 ± 2.0^b^	3.0 ± 0.6^f^	3.7 ± 1.0^b^
0.1% Bioprep + Barapon + Clavodene	5.7 ± 1.3^ab^	34.8 ± 4.8^b^	3.6 ± 0.4^ef^	3.2 ± 1.1^bc^
0.05% Viscozyme + M	5.2 ± 2.3^ab^	27.6 ± 3.8^c^	3.6 ± 0.7^def^	2.9 ± 2.1^bc^
Untreated	3.2 ± 1.5^bc^	42.0 ± 5.5^a^	5.0 ± 0^a^	6.2 ± 1.0^a^

^1^Texazyme BFE from Inotex Ltd., Dvűr Královi, Czech Republic; Multifect Pectinase FE is from Genencor International, Inc., Rochester, NY; Bioprep and Viscozyme L from Novozymes North America, Inc., Franklinton, NC; M is Mayoquest 200 used to provide 18 mM EDTA as chelator.

B + C is Barapon C-108, an amino polycarboxylic acid salt mixture, and Clavodene CIU, a mixture of surfactants (Dexter Chemical L.L.C., Bronx, NY) recommended in cotton scouring. Enzymes and chemicals are used as provided by suppliers and under optimal conditions for activity.

^2^Percent of fiber after passing cleaned fiber through the Shirley Analyzer flax stem. ^3^Modified method ASTM D1445-95, 1999. ^4^ASTM D7025-04a, 2005. ^5^ASTM D7076-05, 2005.

^a,b,c,d,e,f^Values followed by different letters differ at *P* ≤ 0.05.

Data modified from [34].

**Table 11:** Properties of Bioprep-retted and Viscozyme-retted linseed flax varieties.

Retting formulation^1^	Fine fiber yield (%)^2^	Strength (g/tex)^3^	Elongation (%)^3^	Fineness^4^
Hermes Bioprep; M	5.9 ± 0.3^bc^	36.7 ± 0.9^a^	1.9 ± 0.21^a^	4.1 ± 0.2^a^
Hermes seed flax Viscozyme + M	5.0 ± 0.6^c^	21.3 ± 1.8^c^	1.4 ± 0.1^b^	3.0 ± 0.1^b^
Omega seed flax Bioprep; M	8.4 ± 0.3^a^	30.5 ± 0.1^b^	2.0 ± 0.1^a^	1.1 ± 0.1^c^
Omega seed flax Viscozyme + M	6.3 ± 0^b^	20.7 ± 1.5^c^	1.1 ± 0.1^c^	1.2 ± 0^c^

^1^Bioprep and Viscozyme used at under optical conditions for the enzymes. M is Mayoquest 200 used to provide 18 mM EDTA as chelator. In use with Bioprep, incubated with M follows after enzyme soak. In use with Viscozyme, M is used with enzyme mixture.

^2^Percent of fiber after passing cleaned fiber through the Shirley Analyzer.

^3^Modified test method ASTM D1445-95, 1999.

^4^Airflow method.

^a,b,c^Values followed by different letters differ at *P* ≤ 0.05.

Data modified from [35].

**Table 12:** Bioprep-retted Hermes seed flax to optimize retting conditions.

Retting formulation^1^	Fine fiber yield (%)^3^	Strength (g/tex)^4^	Fineness^5^	Predicted shive (%)^6^
0.1% followed by M	10.0 ± 2.9^bcd^	34.7 ± 2.1^a^	4.5 ± 0.1^ab^	5.1 ± 1.9^bc^
0.1% containing M	6.3 ± 0.2^ef^	31.8 ± 1.5^a^	4.5 ± 0.1^ab^	4.6 ± 0.9^bc^
0.1% no chelator	5.6 ± 0.4^f^	ND	ND	11.4 ± 2.6^a^
0.5% followed by M	10.7 ± 2.5^bcd^	36.1 ± 3.6^a^	4.5 ± 0.1^ab^	2.0 ± 1.1^def^
0.5% containing M	9.3 ± 2.7^bcdef^	33.3 ± 1.4^a^	4.5 ± 0.1^a^	3.9 ± 1.9^bcd^
0.5% no chelator	8.5 ± 1.0^cdef^	ND	ND	5.7 ± 1.7^b^
1.0.% followed by M	11.8 ± 2.2^abc^	32.1 ± 0.7^a^	4.3 ± 0.1^c^	1.7 ± 0.9^ef^
1.0% containing M	9.0 ± 1.3^bcdef^	30.6 ± 1.1^a^	4.4 ± 0.1^abc^	3.6 ± 1.4^bcdef^
1.0% no chelator	9.5 ± 0.3^bcde^	ND	ND	3.7 ± 1.5^bcde^
1.5% followed by M	11.8 ± 2.1^abc^	29.8 ± 6.8^a^	4.1 ± 0.1^d^	1.5 ± 0.2^f^
1.5% containing M	8.1 ± 0.1^def^	33.2 ± 0.6^a^	4.4 ± 0.1^abc^	3.0 ± 1.1^cdef^
1.5% no chelator	ND	ND	ND	ND
2.0% followed by M	10.2 ± 2.0^bcd^	32.6 ± 0.9^a^	4.1 ± 0.1^d^	2.3 ± 1.2^def^
2.0% containing M	7.4 ± 1.1^def^	31.6 ± 0.6^a^	4.4 ± 0.1^bc^	2.9 ± 1.3^cdef^
2.0% no chelator	ND	ND	ND	ND
5.0% followed by M^2^	11.7 ± 2.9^abc^	32.6 ± 1.3^a^	4.2 ± 0.1^de^	1.6 ± 0.5^ef^
5.0% containing M^2^	13.2 ± 5.2^a^	33.9 ± 0.8^a^	4.1 ± 0.1^d^	2.0 ± 0.4^def^
5.0% no chelator	12.7 ± 2.4^ab^	29.8 ± 3.6^a^	4.2 ± 0.1^de^	2.3 ± 0.8^def^

^1^Hermes flax was dried at 55°C before crimping through the 9-roller calender. M is 1.83% Mayoquest 200. Triplicate samples of 150 g were tested.

^2^Mayoquest used at 3.0% (30 mM EDTA) as chelator (C).

^3^Shirley-cleaned fiber yield after 1 pass.^3^Modified test method ASTM D1445-95, 1999.

^4^Stelometer. ^5^Airflow. ^6^ASTM D7076-05, 2005.

^a,b,c,d,e,f^Values followed by different letters differ at *P* ≤ 0.05.

Data from [35].

**Table 13:** Properties of 50 : 50 flax : cotton blended yarns after atomized enzyme treatment.

Treatment	Single end strength (g/tex)	Elongation (%)	Strength CV (%)	Neps/250 yards	Thick places/250 yards	Thin places/250 yards	Irregularity CV (%)
Untreated	10.85	5.73	8.98	22	573	138	18.2
Buffer	7.32	5.90	11.33	17	235	64	20.8
Lipase	8.49	6.65	10.8	8	150	23	18.0
Arabinase	8.50	5.95	7.55	9	137	34	18.4
Xylanase	8.24	5.70	9.00	6	175	66	19.7
Cellulase	7.39	6.33	13.71	24	230	40	22.2

Data modified from [14, 36].

## References

[1] Borland V. S. (2002). *From Flower to Fabric*.

[2] Tortora P. G., Collier B. J. (1997). *Understanding Textiles*.

[3] Hamilton I. T. (1986). Linen. *Textiles*.

[4] Franck R. R., Sharma H. S. S., van Sumere C. F. (1992). The history and present position of linen. *The Biology and Processing of Flax*.

[5] Pfefferkorn R. (1944). *Oregon Fiber Flax for an American Linen Industry*.

[6] Stephens G. R. (1996). Connecticut fiber flax trials. *Bulletin*.

[7] Robinson B. B., Hutcheson T. B. (1932). *Circular*.

[8] Hurst W. M., Nelson E. G., Harmond J. E., Klein L. M., Fishler D. W. (1953). *Station Bulletin*.

[9] Kozlowski R. Producing for the market.

[10] Kozlowski R. (2011). Euroflax newsletter.

[11] Machkiewicz M., Barriga-Bedoya J., Mankowski J., Pniewska I. Global flax market situation. Fiber foundations—transportation, clothing, and shelter in the bioeconomy.

[12] Dodes R. Hemmed in by cotton, Hanes eases into flax.

[13] Rodie J. B. Flax unshackled.

[14] McAlister D. D., Foulk J. A., Akin D. E., Annis P. A. Cotton fibres: proportion and interaction with flax fibres in blends: focus on rotor spun yarn.

[15] Berglund D. R., Janick J., Whipkey A. (2002). Flax: new uses and demands. *Trends in New Crops and New Uses*.

[17] Carter J. F. (1993). Potential of flaxseed and flaxseed oil in baked goods and other products in human nutrition. *Cereal Foods World*.

[18] Akin D. E. (2012). Flax fiber. *Kirk-Othmer Encyclopedia of Chemical Technology*.

[19] Fiber foundations—transportation, clothing and shelter in the bioeconomy.

[20] Hänninen T., Hughes M., Müssig J. (2010). Historical, contemporary and future applications. *Industrial Applications of Natural Fibres*.

[21] Nandy S., Rowland G. G. Dual purpose flax (*Linum usitatissimum* L.) improvement using anatomical and molecular approaches, fiber foundations—transportation, clothing and shelter in the bioeconomy.

[22] Dormier K. W. An overview of the flax fibre industry in North America.

[23] Morrison W. H., Akin D. E. (2001). Chemical composition of components comprising bast tissue in flax. *Journal of Agricultural and Food Chemistry*.

[24] Akin D. E., Gamble G. R., Morrison W. H., Rigsby L. L., Dodd R. B. (1996). Chemical and structural analysis of fiber and core tissues from flax. *Journal of the Science of Food and Agriculture*.

[25] Morrison W. H., Akin D. E., Himmelsbach D. S., Gamble G. R. (1999). Chemical, microscopic, and instrumental analysis of graded flax fibre and yarn. *Journal of the Science of Food and Agriculture*.

[26] Akin D. E., Morrison W. H., Gamble G. R., Rigsby L. L., Henriksson G., Eriksson K. E. L. (1997). Effect of retting enzymes on the structure and composition of flax cell walls. *Textile Research Journal*.

[27] Morrison W. H., Archibald D. D., Sharma H. S. S., Akin D. E. (2000). Chemical and physical characterization of water- and dew-retted flax fibers. *Industrial Crops and Products*.

[28] Akin D. E., Morrison W. H., Rigsby L. L., Dodd R. B. (2001). Plant factors influencing enzyme retting of fiber and seed flax. *Journal of Agricultural and Food Chemistry*.

[29] Chen C. C., Adolphson R., Dean J. F. D., Eriksson K. E. L., Adams M. W. W., Westpheling J. (1997). Release of lignin from kraft pulp by a hyperthermophilic xylanase from *Thermatoga maritima*. *Enzyme and Microbial Technology*.

[30] Akin D. E., Slomczynski D., Rigsby L. L., Eriksson K. E. L. (2002). Retting flax with endopolygalacturonase from *Rhizopus oryzae*. *Textile Research Journal*.

[31] Akin D. E., Rigsby L. L., Perkins W. (1999). Quality properties of flax fibers retted with enzymes. *Textile Research Journal*.

[32] Akin D. E., Foulk J. A., Dodd R. B. (2002). Influence on flax fibers of components in enzyme retting formulations. *Textile Research Journal*.

[33] Akin D. E., Foulk J. A., Dodd R. B., McAlister D. D. (2001). Enzyme-retting of flax and characterization of processed fibers. *Journal of Biotechnology*.

[34] Foulk J. A., Akin D. E., Dodd R. B. (2008). Influence of pectinolytic enzymes on retting effectiveness and resultant fiber properties. *BioResources*.

[35] Akin D. E., Condon B., Sohn M., Foulk J. A., Dodd R. B., Rigsby L. L. (2007). Optimization for enzyme-retting of flax with pectate lyase. *Industrial Crops and Products*.

[36] Evans J. D., Akin D. E., Morrison W. H., Himmelsbach D. S., McAlister D. D., Foulk J. A. (2003). Modifying dew-retted flax fibers and yarns with a secondary enzymatic treatment. *Textile Research Journal*.

[37] van Sumere C. F., Sharma H. S. S., van Sumere C. F. (1992). Retting of flax with special reference to enzyme-retting. *The Biology and Processing of Flax*.

[38] Akin D. E., Müssig J. (2010). Flax—structure, chemistry, retting, and processing. *Industrial Applications of Natural Fibres*.

[39] Stern K. R., Jansky S., Bidlack J. E. (2003). *Introductory Plant Biology*.

[40] Akin D. E., Rigsby L. L., Morrison W. H. (2004). Oil red as a histochemical stain for natural fibers and plant cuticle. *Industrial Crops and Products*.

[41] Achwal W. B., Roy N. (1985). Rapid staining test to assess residual wax in pretreated cotton fabrics.

[42] Himmelsbach D. S., Khahili S., Akin D. E. (1999). Near-infrared—fourier-transform—Raman microspectroscopic imaging of flax stems. *Vibrational Spectroscopy*.

[43] Henriksson G., Akin D. E., Hanlin R. T. (1997). Identification and retting efficiencies of fungi isolated from dew-retted flax in the United States and Europe. *Applied and Environmental Microbiology*.

[44] Akin D. E., Rigsby L. L., Henriksson G., Eriksson K. E. L. (1998). Structural effects on flax stems of three potential retting fungi. *Textile Research Journal*.

[45] Henriksson G., Akin D. E., Rigsby L. L., Patel N., Eriksson K. E. L. (1997). Influence of chelating agents and mechanical pretreatment on enzymatic retting of flax. *Textile Research Journal*.

[46] Morrison W. H., Holser R., Akin D. E. (2006). Cuticular wax from flax processing waste with hexane and super critical carbon dioxide extractions. *Industrial Crops and Products*.

[47] Holser R. A., Akin D. E. (2008). Extraction of lipids from flax processing waste using hot ethanol. *Industrial Crops and Products*.

[48] Sarkanen K. V., Ludwig C. H., Sarkanen K. V., Ludwig C. H. (1971). Definition and nomenclature. *Lignins: Occurrence, Formation, Structure, and Reactions*.

[49] Ross K., Mazza G. (2010). Characteristics from flax shives as affected by extraction conditions. *International Journal of Molecular Science*.

[50] Blanchette R. A., Krueger E. W., Haight J. E., Akhtar M., Akin D. E. (1997). Cell wall alterations in loblolly pine wood decayed by the white-rot fungus, *Ceriporiopsis subvermispora*. *Journal of Biotechnology*.

[51] Marshall W. E., Wartelle L. H., Akin D. E. (2007). Flax shive as a source of activated carbon for metals remediation. *BioResources*.

[52] Klasson K. T., Wartelle L. H., Lima I. M., Marshall W. E., Akin D. E. (2009). Activated carbons from flax shive and cotton gin waste as environmental adsorbents for the chlorinated hydrocarbon trichloroethylene. *Bioresource Technology*.

[53] Kim J. W., Mazza G. (2009). Extraction and separation of arbohydrates and phenolic compounds in flax shives with pH-controlled pressurized low polarity water. *Journal of Agricultural and Food Chemistry*.

[54] Sohn M., Barton F. E., Morrison W. H., Akin D. E. (2004). Prediction of shive content in pilot plant processed flax by near infrared reflectance spectroscopy. *Journal of Near Infrared Spectroscopy*.

[55] Sohn M., Barton F. E., Akin D. E., Morrison W. H. (2004). A new approach for estimating purity of processed flax fibre by NIR spectroscopy. *Journal of Near Infrared Spectroscopy*.

[56] Akin D. E. (2005). Standards for flax fiber. *Standardization News*.

[57] Akin D. E., Müssig J. M. (2010). Flax—ASTM standardization and harmonization. *Industrial Applications of Natural Fibres*.

[58] de Haseth J. A., Akin D. E., Barton F. E. Sensors and chemometrics for flax fiber quality and for processing, fiber foundations—transportation, clothing and shelter in the bioeconomy.

[59] Barton F. E., Akin D. E., Morrison W. H., Ulrich A., Archibald D. D. (2002). Analysis of fiber content in flax stems by near-infrared spectroscopy. *Journal of Agricultural and Food Chemistry*.

[60] Khalili S., Akin D. E., Pettersson B., Henriksson G. (2002). Fibernodes in flax and other bast fibers. *Journal of Applied Botany*.

[61] Peters R. H. (1963). The chemistry of fibers. *Textile Chemistry*.

[62] Buschle-Diller G., Zeronian S. H., Pan N., Yoon M. Y. (1994). Enzymatic hydrolysis of cotton, linen, ramie, and viscose rayon fabrics. *Textile Research Journal*.

[63] Focher B., Marzetti A., Sharma H. S. S., Sharma H. S. S., van Sumere C. F. (1992). Changes in the structure and properties of flax fibre during processing. *The Biology and Processing of Flax*.

[64] Bos H. L., van den Oever M. J. A., Peters O. C. J. J. (2002). Tensile and compressive properties of flax fibres for natural fibre reinforced composites. *Journal of Materials Science*.

[65] Akin D. E., Henriksson G., Evans J. D., Adamsen A. P. S., Foulk J. A., Dodd R. B. (2004). Progress in enzyme-retting of flax. *Journal of Natural Fibers*.

[66] Morvan C., Andème-Onzighi C., Girault R., Himmelsbach D. S., Driouich A., Akin D. E. (2003). Building flax fibres: more than one brick in the walls. *Plant Physiology and Biochemistry*.

[67] Gorshkova T. A., Wyatt S. E., Salnikov V. V. (1996). Cell-wall polysaccharides of developing flax plants. *Plant Physiology*.

[68] Stewart D., McDougall G. J., Baty A. (1995). Fourier-transform infrared microspectroscopy of anatomically different cells of flax (*Linum usitatissimum*) stems during development. *Journal of Agricultural and Food Chemistry*.

[69] Girault R., His I., Andeme-Onzighi C., Driouich A., Morvan C. (2000). Identification and partial characterization of proteins and proteoglycans encrusting the secondary cell walls of flax fibres. *Planta*.

[70] Gorshkova T. A., Salnikov V. V., Pogodina N. M. (2000). Composition and distribution of cell wall phenolic compounds flax (*Linum usitatissimum* L.) stem tissues. *Annals of Botany*.

[71] Love G. D., Snape C. E., Jarvis M. C., Morrison I. M. (1994). Determination of phenolic structures in flax fibre by solid-state ^13^C NMR. *Phytochemistry*.

[72] Morrison W. H., Himmelsbach D. S., Akin D. E., Evans J. D. (2003). Chemical and spectroscopic analysis of lignin in isolated flax fibers. *Journal of Agricultural and Food Chemistry*.

[73] Sharma H. S. S., van Sumere C. F. (1992). Enzyme treatment of flax. *The Genetic Engineer and Biotechnologist*.

[74] Gamble G. R., Snook M. E., Henriksson G., Akin D. E. (2000). Phenolic constituents in flax bast tissue and inhibition of cellulase and pectinase. *Biotechnology Letters*.

[75] Bandyopadhyay-Ghosh S., Ghosh S. B., Sain M., Müssig J. (2010). Cellulose nanocomposites. *EdIndustrial Applications of Natural Fibres*.

[76] Akin D. E., Müssig J. Chemistry of plant fibres. *Industrial Applications of Natural Fibres*.

[77] Sakai T., Sakamoto T., Hallaert J., Vandamme E. J. (1993). Pectin, pectinase, and protopectinase: production, properties, and applications. *Advances in Applied Microbiology*.

[78] Davis E. A., Derouet C., du Penhoat C. H., Morvan C. (1990). Isolation and an N.M.R. study of pectins from flax (*Linum usitatissimum* L.). *Carbohydrate Research*.

[79] Meijer W. J. M., Vertregt N., Rutgers B., van de Waart M. (1995). The pectin content as a measure of the retting and rettability of flax. *Industrial Crops and Products*.

[80] Sharma H. S. S. (1988). Chemical retting of flax using chelating compounds.

[81] Himmelsbach D. S., Khalili S., Akin D. E. (1998). FT-IR microspectroscopic imaging of flax (*Linum usitatissimum* L.) stems. *Cellular and Molecular Biology*.

[82] Andeme-Onzighi C., Girault R., His I., Morvan C., Driouich A. (2000). Immunocytochemical characterization of early-developing flax fiber cell walls. *Protoplasma*.

[83] His I., Andème-Onzighi C., Morvan C., Driouich A. (2002). Microscopic studies on mature flax fibers embedded in LR White: immunogold localization of cell wall matrix polysaccharides. *Journal of Histochemistry and Cytochemistry*.

[84] Jauneau A., Quentin M., Driouich A. (1997). Micro-heterogeneity of pectins and calcium distribution in the epidermal and cortical parenchyma cell walls of flax hypocotyl. *Protoplasma*.

[85] Jauneau A., Cabin-Flaman A., Verdus M. C., Ripoll C., Thellier M. (1994). Involvement of calcium in the inhibition of endopolygalacturonase. *Plant Physiology and Biochemistry*.

[86] Ansari I. A., East G. C., Johnson D. J. (1990). Structure-property relationships in natural cellulosic fibres. Part I: characterisation. *Journal of the Textile Institute*.

[87] Brown A. E., Sharma H. S. S., Black D. L. R. (1986). Relationships between pectin content of stems of flax cultivars, fungal cell wall-degrading enzymes and pre-harvest retting. *Annals of Applied Biology*.

[88] Bochek A. M., Zabivalova N. M., Shamolina I. I., Grishanov S. A. (2002). Isolation of pectins from flax pedicels and fibers and their characterization. *Russian Journal of Applied Chemistry*.

[89] Sultana C., Sharma H. S. S., van Sumere C. F. (1992). Scutching of retted-flax straw. *The Biology and Processing of Flax*.

[90] Ross T., Sharma H. S. S., van Sumere C. F. (1992). Preparing and spinning of flax fibre. *The Biology and Processing of Flax*.

[91] Daenekindt A. (2004). Flax, hemp, and allied fibres in the world. *Euroflax Newsletter*.

[92] Sharma H. S. S., Faughey G. J. (1999). Comparison of subjective and objective methods to assess flax straw cultivars and fibre quality after dew-retting. *Annals of Applied Biology*.

[93] Brown A. E. (1984). Epicoccum nigrum, a primary saprophyte involved in the retting of flax. *Transactions of the British Mycological Society*.

[94] Easson D. L., Long E. N. J., Sharma H. S. S., van Sumere C. F. (1992). Pre-harvest retting of flax with glyphosate. *The Biology and Processing of Flax*.

[95] Goodman A. M., Ennos A. R., Booth I. (2002). A mechanical study of retting in glyphosate treated flax stems (*Linum usitatissimum*). *Industrial Crops and Products*.

[96] Sharma H. S. S. (1986). The role of bacteria in retting of desiccated flax during damp weather. *Applied Microbiology and Biotechnology*.

[97] Horne M., Harwood R., McCormick P., Harwood J. The commercial production of short-fibre flax for cottonization, fiber foundations—transportation, clothing, and shelter in the bioeconomy.

[98] Dujardin A. *Vlas-roten*.

[99] Henriksson G., Eriksson K. E. L., Kimmel L., Akin D. E. (1998). Chemical/physical retting of flax using detergent and oxalic acid at high pH. *Textile Research Journal*.

[100] Sharma H. S. S. (1987). Studies on chemical and enzyme retting of flax on a semi-industrial scale and analysis of the effluents for their psycho-chemical components. *International Biodeterioration*.

[101] van den Oever M. J. A., Bas N., van Soest L. J. M., Melis C., van Dam J. E. G. (2003). Improved method for fibre content and quality analysis and their application to flax genetic diversity investigations. *Industrial Crops and Products*.

[102] Costard H. Process for treating sclerenchyma fibers, in particular flax.

[103] Zimmer H., Kloss K. D. Ultrasonic break down of hemp.

[104] Tubach M., Kessler R. W. Interdisciplinary approach for new flax products: examples of applied research at the IAF.

[105] Sotton M., Ferrari M. (1989). Le lin ultra-affine par le traitement hydrolyse flash. *L’Industrie Textile*.

[106] Kessler R. W., Becker U., Kohler R., Goth B. (1998). Steam explosion of flax—a superior technique for upgrading fibre value. *Biomass and Bioenergy*.

[107] Yachmenev V. G., Blanchard E. J., Lambert A. H. (1998). Use of ultrasonic energy in the enzymatic treatment of cotton fabric. *Industrial and Engineering Chemistry Research*.

[108] van Sumere C. F., Sharma H. S. S. (1991). Analyses of fine flax fibre produced by enzymatic retting. *Aspects of Applied Biology*.

[109] Sharma H. S. S. (1987). Screening of polysaccharide-degrading enzymes for retting flax stem. *International Biodeterioration*.

[110] Sharma H. S. S., Lefevre J., Boucaud J., Sharma H. S. S., van Sumere C. F. (1992). Role of microbial enzymes during retting and their effect on fibre characteristics. *The Biology and Processing of Flax*.

[111] Sharma H. S. S., van Sumere C. F. (1992). *The Biology and Processing of Flax*.

[112] Henriksson G., Akin D. E., Slomczynski D., Eriksson K. E. L. (1999). Production of highly efficient enzymes for flax retting by *Rhizomucor pusillus*. *Journal of Biotechnology*.

[113] Adamsen A. P. S., Akin D. E., Rigsby L. L. (2002). Chelating agents and enzyme retting of flax. *Textile Research Journal*.

[114] Adamsen A. P. S., Akin D. E., Rigsby L. L. (2002). Chemical retting of flax straw under akaline conditions. *Textile Research Journal*.

[115] ASTM D 1445-95 Standard test method for breaking strength and elongation for cotton fibers (flat bundle method).

[116] ASTM D 1448-90 Standard test method for breaking strength and elongation for cotton fibers.

[117] International Standard 2370

[118] Foulk J. A., Akin D. E., Dodd R. B. (2009). Miniature spinning enzyme-retted flax fibers. *Journal of Natural Fibers*.

[119] Akin D. E., Dodd R. B., Perkins W., Henriksson G., Eriksson K. E. L. (2000). Spray enzymatic retting: a new method for processing flax fibers. *Textile Research Journal*.

[120] Foulk J. A., Akin D. E., Dodd R. B. (2001). Processing techniques for improving enzyme-retting of flax. *Industrial Crops and Products*.

[121] Akin D. E., Morrison W. H., Rigsby L. L., Evans J. D., Foulk J. A. (2003). Influence of water presoak on enzyme-retting of flax. *Industrial Crops and Products*.

[122] Akin D. E., Dodd R. B., Foulk J. A. (2005). Pilot plant for processing flax fiber. *Industrial Crops and Products*.

[123] Akin D. E., Epps H. H., Archibald D. D., Sharma H. S. S. (2000). Color measurement of flax retted by various means. *Textile Research Journal*.

[124] Epps H. H., Akin D. E., Foulk J. A., Dodd R. B. (2001). Color of enzyme-retted flax fibers affected by processing, cleaning, and cottonizing. *Textile Research Journal*.

[125] Durden D. K., Etters J. N., Sarkar A. K., Henderson L. A., Hill J. E. (2001). Advances in commercial biopreparation of cotton with alkaline pectinase. *AATCC Reviews*.

[126] Etters J. N., Sarkar A. K., Henderson L. A., Liu J. (2001). The influence of biopreparation of cotton with alkaline pectinase on dyeing properties. *AATCC Reviews*.

[127] Antonov V., Marek J., Bjelkova M., Smirous P., Fischer H. (2007). Easily available enzymes as natural retting agents. *Biotechnology Journal*.

[128] Marek J., Antonov V., Bjelkova M., Smirous P., Fischer H., Janosik S. Enzymatic bioprocessing—new tool for extensive natural fibre source utilization, fiber foundations—transportation, clothing, and shelter in the bioeconomy.

[129] Evans J. D., Akin D. E., Foulk J. A. (2002). Flax-retting by polygalacturonase-containing enzyme mixtures and effects on fiber properties. *Journal of Biotechnology*.

[130] Zhang J., Henriksson G., Johansson G. (2000). Polygalacturonase is the key component in enzymatic retting of flax. *Journal of Biotechnology*.

[131] Himmelsbach D. S., Khalili S., Akin D. E. (2002). The use of FT-IR microspectroscopic mapping to study the effects of enzymatic retting of flax (*Linum usitatissimum* L) stems. *Journal of the Science of Food and Agriculture*.

[132] Zhang J., Johansson G., Pettersson B. (2003). Effects of acidic media pre-incubation on flax enzyme retting efficiency. *Textile Research Journal*.

[133] Brühlmann F., Leupin M., Erismann K. H., Fiechter A. (2002). Enzymatic degumming of ramie bast fibers. *Journal of Biotechnology*.

[134] Rho D., Yang J., Lorrain M. -J. Processing of flax fibers for biocomposites using a thermostable pectate lyase, fiber foundations—transportation, clothing, and shelter in the bioeconomy.

[135] Foulk A. ., Rho D., Alcock M. M., Ulven C. A., Huo S. (2011). Modifications caused by enzyme-retting and their effect on biocomposite performance. *Advances in Materials Science and Engineereing*.

[136] Hu W., Ton-That M.-T., Denault J., Rho D., Yang J., Lau P. C. K. (2012). Comparison between dew-retted and enzyme-retted flax fibers as reinforcing material for composites. *Polmer Engineering and Science*.

[137] Ekblad C., Pettersson B., Zhang J., Jernberg S., Henriksson G. (2005). Enzymatic-mechanical pulping of bast fibers from flax and hemp. *Cellulose Chemistry and Technology*.

[138] Evans J. D., Akin D. E., Morrison W. H., Himmelsbach D. S., Foulk J. A. (2002). Modifying dew-retted flax fibers by means of an air-atomized enzyme treatment. *Textile Research Journal*.

[139] Fischer H., Müssig J., Bluhm C. (2006). Enzymatic modification of hemp fibres for sustainable production of high quality materials: influence of processing parameters. *Journal of Natural Fibers*.

[140] Morrison W. H., Akin D. E., Ramaswamy G., Baldwin B. (1996). Evaluating chemically retted kenaf using chemical, histochemical, and microspectrophotometric analyses. *Textile Research Journal*.

[141] Ramaswamy G. N., Ruff C. G., Boyd C. R. (1994). Effect of bacterial and chemical retting on kenaf fiber quality. *Textile Research Journal*.

[142] Akin D. E., Sethuraman A., Morrison W. H., Martin S. A., Eriksson K. E. L. (1993). Microbial delignification with white rot fungi improves forage digestibility. *Applied and Environmental Microbiology*.

[143] Akin D. E., Morrison W. H., Rigsby L. L., Gamble G. R., Sethuraman A., Eriksson K. E. L. (1996). Biological delignification of plant components by the white rot fungi *Ceriporiopsis subvermispora* and *Cyathus stercoreus*. *Animal Feed Science and Technology*.

